# Fine-tuning BACH2 dosage balances stemness and effector function to enhance antitumor T cell therapy

**DOI:** 10.1038/s41590-025-02389-z

**Published:** 2026-01-16

**Authors:** Alberto G. Conti, Alexander C. Evans, Teresa von Linde, Christian Deo T. Deguit, Sarah K. Whiteside, Alexander J. Wesolowski, Charlotte J. Imianowski, Yumi Yamashita-Kanemaru, Layla Dahmani, Jack Chapman, Ardon M. Pillay, Aws Al-Deka, Randy Greaves, Oliver Burton, Panagiota Vardaka, Shienny Sampurno, Iván Pérez-Núñez, Nicole Y. L. Saw, Jie Yang, Andrew J. M. Howden, Klaus Okkenhaug, Suman Mitra, Bartlomiej Swiatczak, Ian A. Parish, Rahul Roychoudhuri

**Affiliations:** 1 Department of Pathology, University of Cambridge, Cambridge, UK; 2 Cancer Immunology Program, Peter MacCallum Cancer Centre, Melbourne, Victoria Australia; 3 Sir Peter MacCallum Department of Oncology, The University of Melbourne, Parkville, Victoria Australia; 4 Department of Oncology, University of Oxford, Oxford, UK; 5 Centre for Immuno-Oncology, Nuffield Department of Medicine, University of Oxford, Oxford, UK; 6 Division of Cell Signalling and Immunology, School of Life Sciences, University of Dundee, Dundee, UK; 7 Université de Lille, CNRS, Inserm, CHU Lille, UMR9020-U1277 CANTHER, Lille, France

**Keywords:** Tumour immunology, Immunotherapy, Cancer immunotherapy, CD8-positive T cells

## Abstract

Adoptive T cell therapies are limited by poor persistence of transferred cells. Attempts to enhance persistence have focused on genetic induction of constitutively hyperactivated but potentially oncogenic T cell states. Physiological T cell responses are maintained by quiescent stem-like/memory cells dependent upon the transcription factor BACH2. Here we show that quantitative control of BACH2 dosage regulates differentiation along the continuum of stem and effector CD8⁺ T cell states, enabling engineering of synthetic states with persistent antitumor activity. While conventional high-level overexpression of BACH2 enforces quiescence and hinders tumor control, low-dose BACH2 expression promotes persistence without compromising effector function, enhancing anticancer efficacy. Mechanistically, low-dose BACH2 partially attenuates Jun occupancy at highly AP-1-dependent genes, restraining terminal differentiation while preserving effector programs. Similarly, dose optimization enables effective deployment of quiescence factor FOXO1. Thus, quantitative control of gene payloads yields qualitative effects on outcome with implications for quiescence factor deployment in cell therapy.

## Abstract (web summary)

One of three back-to-back papers to show dosage of BACH2 can modulate T cell differentiation and function and how we might apply this to enhance chimeric antigen receptor T cell therapies for cancer.

## Main

Maintenance of antigen-specific CD8^+^ T cell responses is essential for immunological memory and durable responses to chronic antigens. Long-term maintenance of T cell responses requires a division of labor between quiescent long-lived progenitor cells and their shorter-lived functional progeny. During chronic antigen exposure, stem-like progenitor-exhausted T (T_PEX_) cells, characterized by expression of the transcription factor TCF1 and the cell surface receptor Slamf6, self-renew while giving rise to more functional but shorter-lived intermediate-exhausted T (T_INT_) cells and terminally exhausted T (T_TEX_) cells, the latter characterized by expression of cell surface receptors TIM-3 and CD69 (refs. ^1–3^). We now understand that maintenance of tumor-reactive CD8^+^ T cells is a prerequisite for effective cancer immunotherapy responses. For instance, during checkpoint inhibitor therapy, the relative abundance of T_PEX_ to T_TEX_ cells is associated with improved response to anti-programmed death 1 (PD-1) therapy, with T_PEX_ cells proliferating and giving rise to functional effector cells upon release from inhibitory PD-1 signaling^4,5^.

The efficacy of T cell therapies, including chimeric antigen receptor (CAR) T cell and tumor-infiltrating lymphocyte (TIL) therapy, is also dependent upon optimal persistence. The presence of stem-like T cells within the pre-infusion product associates with improved antitumor responses in both preclinical and clinical settings^6–9^. Moreover, the persistence of CAR T cells is associated with improved clinical responses in certain hematological malignancies^4,6,10,11^ and solid cancers^12^. This fundamental relationship between T cell persistence and therapeutic efficacy underscores the importance of understanding and enhancing the maintenance of T cell therapy responses in cancer.

Several approaches have been used to enhance the persistence of T cell therapy responses. The use of cytokines or small molecules during ex vivo culture, including AKT inhibitors (for example, AKTi-1/2)^13,14^ or bromodomain inhibitors (for example, JQ1)^15^, restrains T cell differentiation during T cell expansion and leads to improved expansion capacity upon adoptive transfer. However, such approaches lead to transient improvements in T cell phenotype, which are rapidly lost upon adoptive transfer. A different approach has been that of genetically engineering T cells to express proteins that confer enhanced persistence or function. Such attempts have included the overexpression of factors with oncogenic potential, including proto-oncogenes such as JUN^16^ and MYB^17^, constitutively active STAT5 variants^5^ and the CARD11–PIK3R3 oncogenic fusion protein^18^, which induce persistently activated but potentially oncogenic T cell states, raising concerns over their potential to drive T cell therapy-derived lymphomas^19^. Consequently, there is interest in exploiting physiological mechanisms of T cell maintenance to safely enhance T cell persistence and efficacy in the context of adoptive T cell therapy.

The transcription factor BACH2 plays a critical role in the quiescence and maintenance of memory CD8^+^ T cell responses after acute viral infection^20^, and in the differentiation of CD8^+^ T_PEX_ cells during chronic viral infection^21^. BACH2 is a 92-kDa transcriptional repressor of the bZIP transcription factor family^20,22,23^. Within both acute and chronic responses, *Bach2* mRNA is expressed in naive and stem-like central memory/progenitor-exhausted CD8^+^ T cells and is downregulated upon differentiation into terminal effector/terminally exhausted CD8^+^ T cells^20,21^. Within naive and memory CD8^+^ T cells, BACH2 binds to TPA response elements (TREs) within the enhancers of effector-associated genes, where it competes with AP-1 factors for genomic binding^20^. Consequently, BACH2 restricts T cell antigen receptor (TCR)-driven effector programs in naive and memory CD8^+^ T cells enabling retention of the quiescent phenotype, required for long-lived memory responses. Consistent with its role as a quiescence factor, recent reports show that BACH2 functions as a tumor suppressor gene in the context of CAR T cell-derived lymphoma^24,25^. Despite its requisite role in T cell maintenance, the quiescence factor activity of BACH2 has not been exploited to enhance maintenance of T cell therapy responses.

Here, we show that quantitative control of BACH2 dosage establishes the continuum of stem and effector states in CD8⁺ T cells and enables engineering of synthetic cell states with enhanced persistence and antitumor efficacy. While constitutive high-level BACH2 expression prevents terminal differentiation but compromises acquisition of cytotoxic functions, low-level BACH2 allows activated cells to retain stem-like features without loss of effector programs. We demonstrate that this principle extends beyond BACH2 to the memory-associated factor FOXO1. Together, these findings identify dosage control of quiescence factors as a fundamental mechanism governing T cell maintenance and provide a framework for safely extending T cell persistence in therapeutic settings.

## Results

### BACH2 overexpression locks T cells in a quiescent ineffective state

**Fig. 1:**
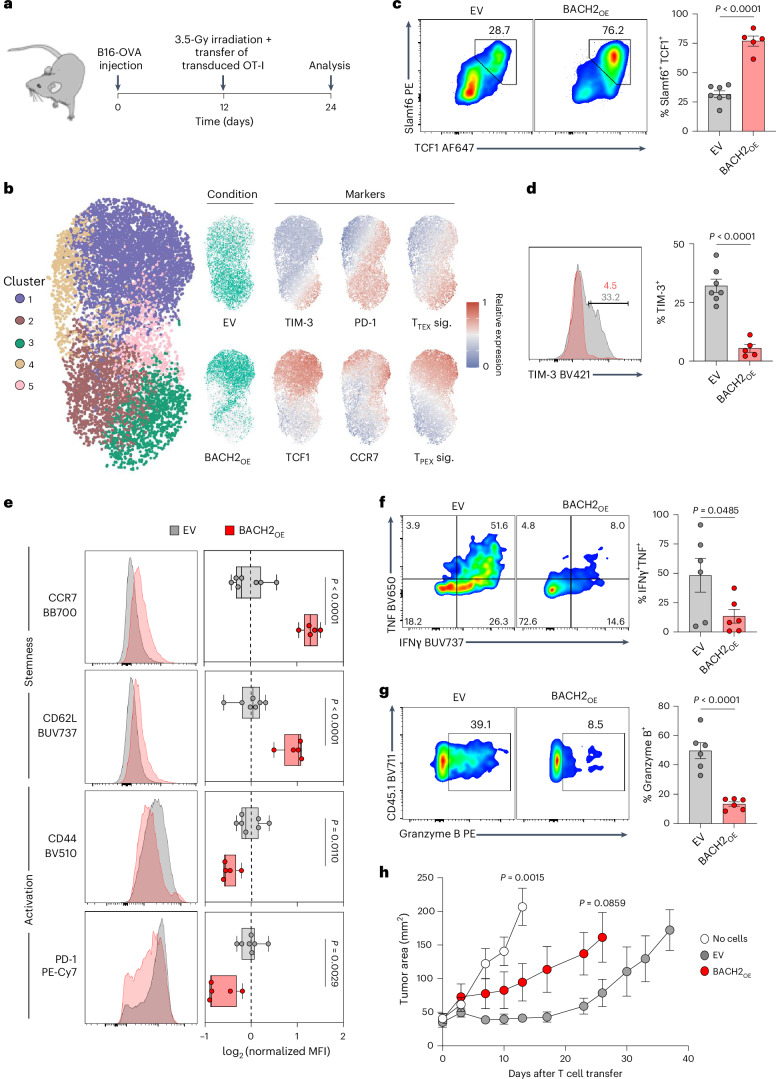
BACH2 overexpression promotes CD8^+^ T cell stemness but limits effector functions. **a**, Experimental schema. Wild-type mice were subcutaneously injected with B16-OVA cells and tumor-bearing mice received 3.5 Gy X-ray irradiation and intravenous injection of 0.5 × 10^6^ OT-I T cells transduced with EV or BACH2_OE_ vectors. **b**, Uniform manifold approximation and projection (UMAP) plot, cluster quantification and protein marker expression from flow cytometry data of EV-transduced and BACH2_OE_-transduced intratumoral OT-I T cells. Phenotypic signatures correspond to the average scaled expression of TIM-3, PD-1 and CD69 (T_TEX_) and TCF1, Slamf6, CD62L and CCR7 (T_PEX_). **c**,**d**, Percentage of Slamf6^+^ TCF1^+^ (**c**) and TIM-3^+^ (**d**) cells in transduced intratumoral OT-I T cells from EV (*n* = 7) or BACH2_OE_ (*n* = 5) and representative flow cytometry plots. **e**, Median fluorescence intensity (MFI) of CCR7, CD62L, CD44 and PD-1 in transduced intratumoral OT-I T cells from EV (*n* = 7) or BACH2_OE_ (*n* = 5), and representative flow cytometry histograms. **f**,**g**, Percentage of IFNγ^+^ TNF^+^ (**f**) and granzyme B^+^ (**g**) cells in transduced intratumoral OT-I T cells from EV (*n* = 6) or BACH2_OE_ (*n* = 6) following ex vivo stimulation with PMA + ionomycin and representative flow cytometry plots. **h**, Tumor measurements of mice injected with B16-OVA and receiving either Hanks’ balanced salt solution (HBSS; no cells, *n* = 6) or OT-I T cells transduced with EV (*n* = 5) or BACH2_OE_ (*n* = 5) as detailed in **a**. Data are representative of two independent experiments with five to eight mice per group in each experiment. Unpaired two-tailed Student’s *t*-test (**b**–**h**). Dots represent independent replicates (**c**–**g**), box plots display the minimum and maximum value (whiskers), median (vertical line) and interquartile range (box) (**e**), bars and errors indicate the mean ± s.e.m. (**c**, **d**, **f** and **g**), and tumor curves represent the mean of independent replicates ± s.e.m. (**h**). Source data

**Extended Data Fig. 1:**
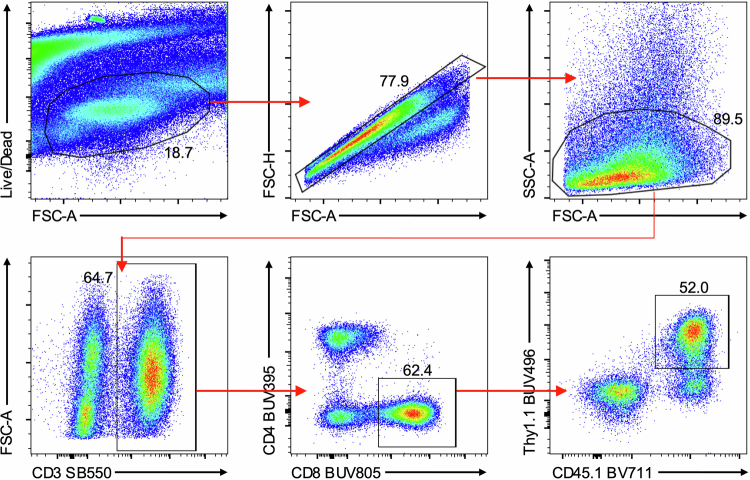
Gating strategy for identification of intratumoral transduced OT-I T cells. Representative gating strategy showing OT-I (CD45.1^+^) transduced (Thy1.1^+^) T cells in B16-OVA tumors.

We and others have shown that BACH2 maintains the pool of stem cell-like memory cells by restricting terminal differentiation in the context of acute and chronic viral infection^20^^,21^. Given the association of stem-like T cells with effective antitumor immune responses, we initially asked whether BACH2 overexpression improves the antitumor efficacy of adoptive T cell therapy. We utilized an adoptive cell therapy model whereby syngeneic B16 melanoma cells expressing the model antigen ovalbumin (B16-OVA) are recognized by OT-I TCR-transgenic CD8^+^ T cells specific for the OVA_257–264_ epitope^26^. OT-I T cells retrovirally transduced with a constitutive BACH2 overexpression (BACH2_OE_) vector or a control empty vector (EV) were adoptively transferred into sublethally irradiated B16-OVA tumor-bearing animals (Fig. 1a). Transduced OT-I T cells were readily identifiable in tumor-bearing recipient animals through expression of the congenic marker CD45.1, and the retroviral transduction marker Thy1.1 (Extended Data Fig. 1).

Flow cytometry revealed a spectrum of differentiation states within tumors. Less differentiated cells were located in cluster 1, including PD-1^+^TCF1^+^TIM-3^−^ T_PEX_ cells, which also expressed other T_PEX_-associated markers such as CCR7. In contrast, cluster 3 contained more highly differentiated cells, characterized by high expression of PD-1 and TIM-3. Other T cell states were distributed among the remaining clusters, such as intermediate PD-1^+^TCF1^−^TIM-3^−^ cells (T_INT_) in clusters 2 and 5, and PD-1^−^TCF1^+^ cells in cluster 4. As anticipated, while EV-transduced cells displayed a continuum of differentiation states within the tumor, BACH2_OE_-transduced cells clustered primarily in cluster 1, corresponding to an induction of a T_PEX_ phenotype^27^ (Fig. 1b). This distribution is consistent with an observed significant increase in the proportion of TCF1^+^Slamf6^+^ cells within the BACH2_OE_ group, as well as a near-complete absence of TIM-3 expression, higher levels of the lymphoid homing receptors CD62L and CCR7 (expressed in naive and memory T cells) and diminished expression of activation markers CD44 and PD-1 (Fig. 1c–e). In addition, BACH2_OE_ severely curtailed production of effector molecules tumor necrosis factor (TNF), interferon gamma (IFNγ) and granzyme B upon 4-h restimulation ex vivo (Fig. 1f,g). Consequently, despite increased expression of markers associated with stemness and reduced levels of terminal differentiation, BACH2-overexpressing OT-I cells mediated impaired antitumor responses compared to EV-transduced cells upon adoptive transfer (Fig. 1h). Collectively, these data suggest that constitutive high-dose overexpression of BACH2 in tumor-targeting T cells locks cells in a memory/progenitor-exhausted state with restricted effector functions, blunting the antitumor efficacy of adoptively transferred CD8^+^ T cells.

### CD8^+^ T_PEX_ cells express intermediate levels of *Bach2*

**Fig. 2:**
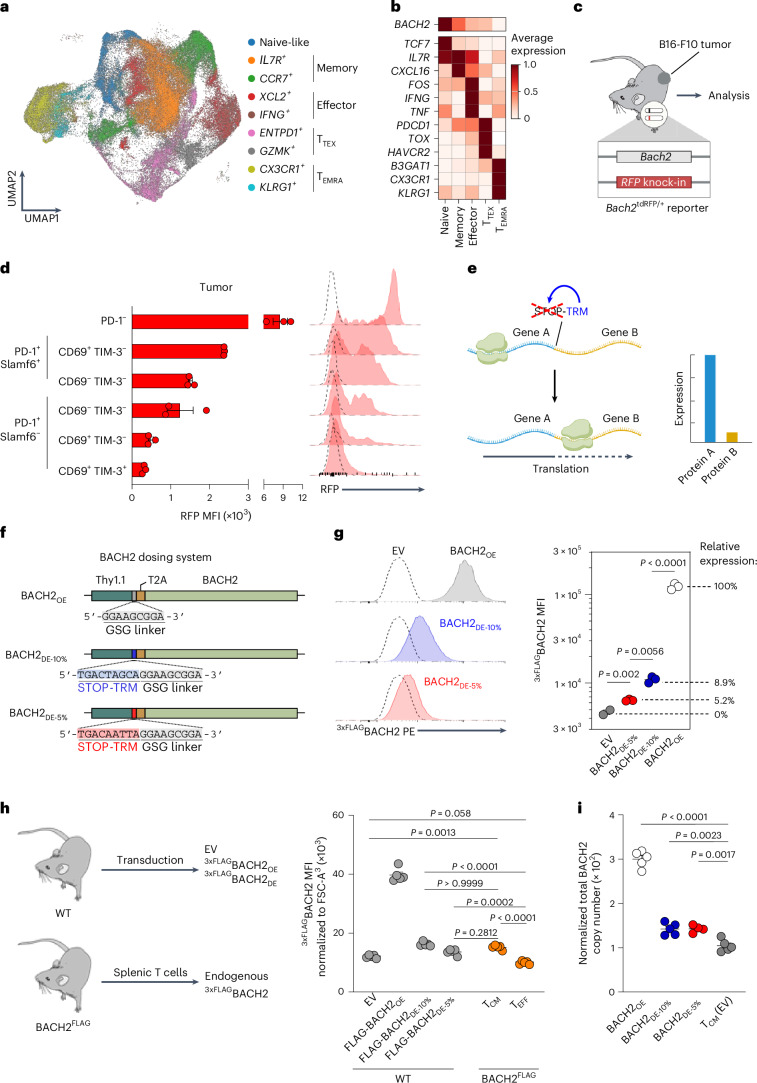
*Bach2* levels in distinct CD8^+^ T cell differentiation states inform development of a *Bach2* dosing system. **a**,**b**, UMAP plot of tumor-infiltrating CD8^+^ T cells from human cancer samples (**a**) and representative marker expression for indicated cluster groups (**b**)^30^. **c**, Schema of *Bach2*^tdRFP/+^ mice for analysis of endogenous *Bach2* regulation. **d**, Histograms of *Bach2*^tdRFP^ expression and frequency of expression from intratumoral CD8^+^ T cell subsets in B16-F10 tumor-bearing mice (*n* = 3). The dashed line represents the signal from a control WT animal. **e**, Diagram depicting the STOP-TRM system used for dosing a payload of interest. Translation of an mRNA by a ribosome will generally be terminated upon encountering a STOP codon. If the STOP codon is flanked by a TRM, termination of translation is partially suppressed, leading to downstream translation at reduced levels of expression^32^. **f**, BACH2_OE_ and BACH2_DE_ vector design. All vectors contained Thy1.1 as a transduction reporter, followed by a glycine-serine-glycine (GSG) linker, a T2A ribosomal skip motif and the *Bach2* ORF tagged in an N-terminal manner with a 3xFLAG tag (^3xFLAG^BACH2). A STOP-TRM was inserted into BACH2_DE_ vectors before the GSG linker to achieve lower levels of *Bach2* expression relative to BACH2_OE_. **g**, MFI of ^3xFLAG^BACH2 expression on OT-I cells transduced with EV (*n* = 2), BACH2_DE_ (*n* = 3) or BACH2_OE_ (*n* = 3) and representative flow cytometry histograms. **h**, Normalized MFI of ^3xFLAG^BACH2 expression in cells derived from either BACH2^FLAG^ or wild-type mice and transduced with the indicated vectors (*n* = 5 for all groups). ^3xFLAG^BACH2 were normalized to forward scatter (FSC-A) to account for variation in cell size. **i**, Normalized copy number of BACH2 from cells transduced with EV (*n* = 5), BACH2_DE-5%_ (*n* = 4), BACH2_DE-5%_ (*n* = 5) and BACH2_OE_ (*n* = 5). Copy number was normalized to total protein mass per cell. Data are representative of two independent experiments (**d**, **g** and **h**). Multiple unpaired two-tailed Student’s *t*-test with Bonferroni correction (**g**). One-way analysis of variance (ANOVA) with Tukey’s or multiple-comparison correction (**h** and **i**). Dots represent independent replicates (**d**, **g**, **h** and **i**), and bars or horizontal lines and error bars indicate the mean ± s.e.m. (**d**, **g**, **h** and **i**). **c** and **e** were created with BioRender.com. Source data

BACH2 is expressed by naive and central memory/progenitor-exhausted CD8^+^ T cells and extinguished upon terminal differentiation^20,21^. However, central memory and progenitor-exhausted CD8^+^ T cells are capable of cytokine polyfunctionality and potent effector function, an observation at odds with the function of BACH2 as a repressor of effector functions^28,29^. To better understand whether a binary *Bach2* expression pattern distinguishes cells in these distinct differentiation states, or whether *Bach2* dosage gradually changes within cells of each state on a per-cell basis, we first analyzed single-cell transcriptional profiles of TILs across various human cancer types^30^. *BACH2* mRNA expression was greatest among naive and memory cells, correlating with *TCF7* (encoding TCF1) and *IL7R* expression (Fig. 2a,b), and decreased progressively with T cell differentiation. Effector cells displayed intermediate levels of *BACH2*, and the lowest levels were observed in *CX3CR1*^+^ and *KLRG1*^*+*^ terminal effector memory reexpressing CD45RA (T_EMRA_) cells. These findings suggested that *BACH2* expression levels are not binary but rather are precisely regulated within CD8^+^ T cells of distinct differentiation states.

**Extended Data Fig. 2:**
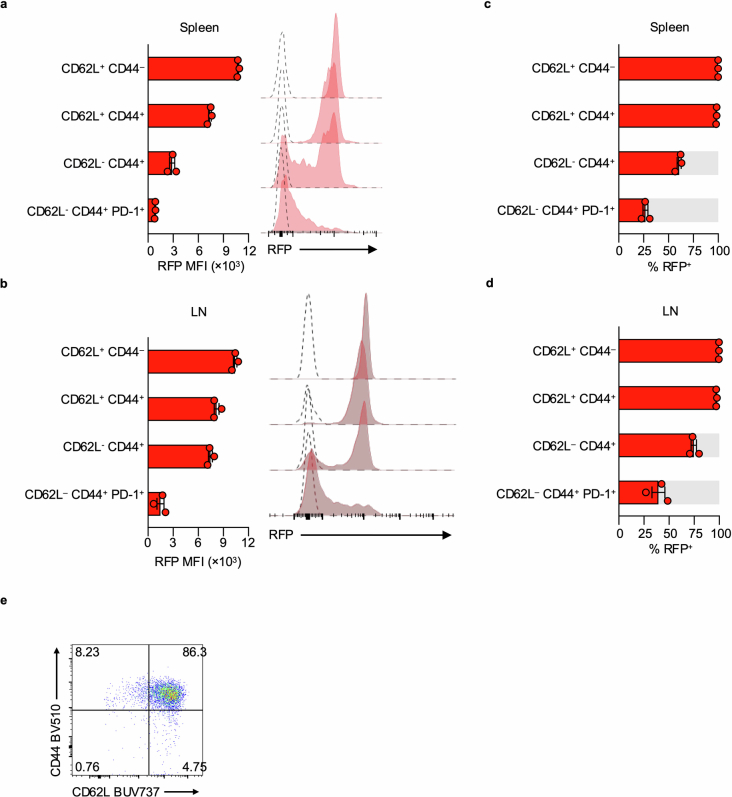
Peripheral CD8 + T cell subsets display distinct Bach2 expression levels. **a-b**, MFI of RFP expression in CD8^+^ T cells from spleen (**a**) and tumor-draining lymph nodes (**b**) of B16-F10 tumor-bearing *Bach2*^tdRFP^ mice, and representative flow cytometry histograms (*n* = 3). **c-d**, Frequency of RFP expression in CD8^+^ T cells from spleen (**c**) and tumor-draining lymph nodes (LN) (**d**) of B16-F10 tumor-bearing *Bach2*^tdRFP^ mice (*n* = 3). **e**, Representative flow cytometry plot of T cells rested in culture for 48 h post-transduction across all vectors. Dots represent independent replicates (**a-d**), bars and error indicate mean ± s.e.m. (**a-d**). Source data

To study *Bach2* expression levels on a per-cell basis with greater resolution, we used *Bach2*^tdRFP^ reporter mice in which a tandem red fluorescent protein (tdRFP) is expressed under the transcriptional control of endogenous *Bach2* regulatory elements^31^ (Fig. 2c). *Bach2*^tdRFP/+^ mice were subcutaneously injected with B16-F10 melanoma cells, and T cells from the tumor, spleen and draining lymph nodes were phenotyped 16 days later. In line with human single-cell RNA-sequencing (scRNA-seq) data, we observed a reduction in the frequency of *Bach2*-positive CD8^+^ T cells as they progressed along both acute and chronic differentiation trajectories, from naive to central memory (T_CM_), effector memory (T_EM_) and effector (T_eff_); and naive to T_PEX_, T_INT_ and T_TEX_, respectively (Fig. 2d and Extended Data Fig. 2a,b). Importantly, when *Bach2* expression was examined on a per-cell basis, we observed graded levels of *Bach2* expression, with intermediate levels in central memory and progenitor-exhausted CD8^+^ T cell subsets (Fig. 2e). CD8^+^ T cells in the spleen and draining lymph nodes displayed comparable *Bach2* expression dynamics, with greatest expression in naive CD62L^+^CD44^−^ cells and lowest expression in antigen-experienced CD62L^−^CD44^+^ cells (Extended Data Fig. 2c,d). Thus, *Bach2* expression levels are progressively downregulated on a per-cell basis, with polyfunctional central memory and progenitor-exhausted cells expressing intermediate levels of *Bach2*.

### Low-dose BACH2 preserves stemness without limiting effector functions

Our experiments showed that endogenous *Bach2* levels are precisely regulated within T cells of distinct differentiation states. Given that constitutive high-dose BACH2 overexpression caused loss of effector functions and antitumor efficacy, we asked whether fine-tuning the level of BACH2 expression would enable programming of a stem-like phenotype without restricting effector function. To test this, we designed a system to enable low-dose expression of BACH2, using mutated translational readthrough motifs (TRMs) to partially attenuate premature translational termination of a *BACH2* open reading frame (ORF) by a stop codon (STOP-TRM; Fig. 2f)^32^. Using two different STOP-TRM mutants, we achieved low-dose expression of BACH2 (BACH2_DE_) at median levels approximately 10% (BACH2_DE-10%_) and 5% (BACH2_DE-5%_) of those achieved by conventional retroviral overexpression, as determined using flow cytometric detection of a 3xFLAG tag at the N terminus of the BACH2 ORF transgene (^3xFLAG^BACH2; Fig. 2g).

To determine how BACH2 expression from our dosing vectors compares to physiological levels, we utilized BACH2^FLAG^ mice, which carry a 3xFLAG tag at the N terminus of the endogenous *Bach2* locus—identical to the tag present in our BACH2 expression constructs^33^. This enabled direct comparison between endogenous and transgenic BACH2 levels using flow cytometry. CD8^+^ T cells from both BACH2^FLAG^ and wild-type mice were transduced with EV, BACH2_OE_ or BACH2_DE_ vectors and rested in culture under identical conditions (Fig. 2h). After 48 h, all groups displayed a central memory phenotype (CD44^+^CD62L^+^; Extended Data Fig. 2e). To account for any differences in cell size, we normalized the 3xFLAG signal to forward scatter. EV-transduced cells from BACH2^FLAG^ mice showed detectable 3xFLAG signal representing endogenous BACH2 levels in central memory cells. Notably, ^3xFLAG^BACH2_DE-5%_-transduced wild-type cells produced 3xFLAG levels comparable to endogenous ^3xFLAG^BACH2 in central memory cells of BACH2^FLAG^ mice, while BACH2_DE-10%_ cells showed slightly higher levels and BACH2_OE_ cells showed a substantially higher signal (Fig. 2h,i). These findings suggest that BACH2_DE_ vectors achieve transgenic BACH2 expression levels similar to those found physiologically in central memory T cells, which express intermediate levels of endogenous BACH2.

To validate these findings using an alternate approach, we performed mass spectrometry (MS)-based quantification of BACH2 protein levels. This analysis showed that BACH2_DE_-transduced cells display a total BACH2 copy number (endogenous + transgenic) normalized to total protein that is comparable and slightly above that from cells in a central memory state (Fig. 2i). Minor distinctions between this result and our 3xFLAG flow cytometry measurements likely reflect that 3xFLAG detection measured only transgene-derived BACH2, whereas MS quantified both endogenous and transgene-derived BACH2, as well as differences in normalization methods.

**Fig. 3:**
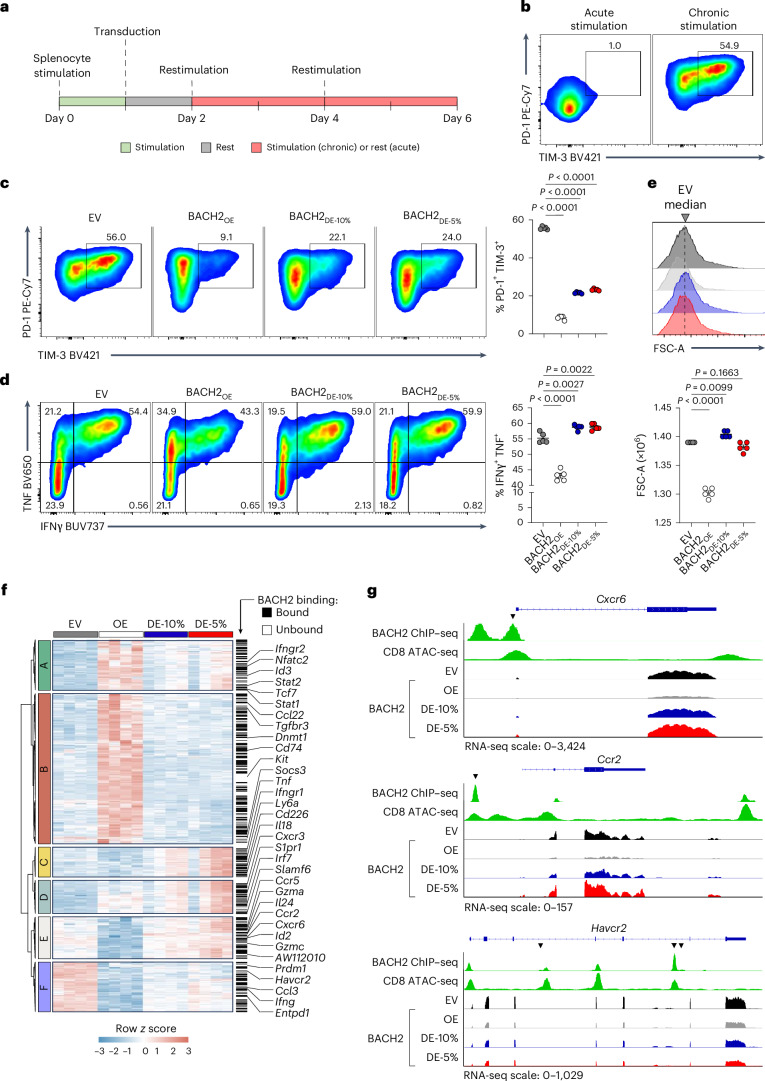
Low-dose expression of BACH2 promotes stemness without compromising effector functions. **a**, Experimental schema. OT-I splenocytes were activated for 24 h with anti-CD3 and anti-CD28 before retroviral transduction. Transduced cells were maintained in IL-2-supplemented media on plates coated with (chronic stimulation) or without (acute stimulation) anti-CD3. **b**, Representative flow cytometry illustrating PD-1 and TIM-3 expression on EV-transduced OT-I cells following acute or chronic stimulation. **c**,**d**, Percentage of PD-1^+^TIM-3^+^ chronically stimulated (**c**) or IFNγ^+^ TNF^+^ acutely stimulated following 4 h of anti-CD3 restimulation in the presence of brefeldin A and monensin (**d**) from OT-I T cells transduced with the indicated vectors (*n* = 5 for all groups) at day 4 and representative flow cytometry plots. **e**, FSC-A of acutely stimulated OT-I T cells transduced with the indicated vectors (*n* = 5 for all groups) at day 4 and representative flow cytometry histograms. **f**, Heat map showing differentially expressed genes (DEGs, *q* < 0.05, log_2_(fold change or FC) > 1) between chronically stimulated transduced OT-I T cells (*n* = 4 for all groups). Color indicates row *z* score. Black bars indicate genes bound by BACH2 based on a prior chromatin immunoprecipitation sequencing (ChIP–seq) analysis^20^. **g**, Alignment showing representative mRNA expression at indicated loci within EV, BACH2_OE_, BACH2_DE-10%_ and BACH2_DE-5%_ of *Cxcr6*, *Ccr2* and *Havcr2*; ChIP–seq analysis of BACH2 binding and ATAC-seq analysis of chromatin accessibility. Black arrowheads represent AP-1 binding motifs (TGA(G/C)TCA) colocalizing with BACH2 binding peaks. Data are representative of three independent experiments (**a**–**e**) with three to five samples per experimental group in each experiment. One-way ANOVA with Dunnett’s multiple-comparison correction (**c**–**e**). Dots represent independent replicates (**c**–**e**); horizontal lines and error bars indicate the mean ± s.e.m (**c**–**e**). Source data

**Extended Data Fig. 3:**
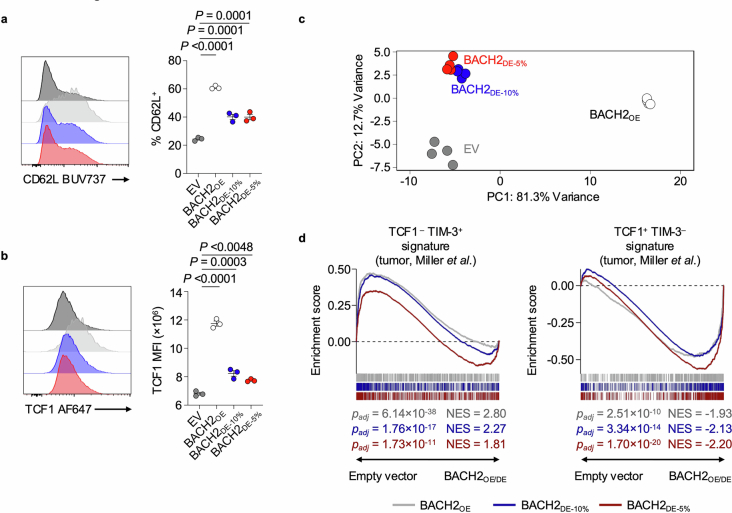
Low-dose expression of BACH2 preserves stemness upon chronic stimulation in vitro. **a-b**, Frequency of CD62L expression (**a**) and TCF1 MFI (**b**) of chronically stimulated transduced OT-I T cells and representative flow cytometry plots (*n* = 3 for all groups). **c**, PCA plot of chronically stimulated transduced OT-I cells on day 6 (*n* = 4 for all groups). **d**, Comparison between EV and BACH2_OE_, BACH2_DE-10%_ or BACH2_DE-5%_ in chronically stimulated transduced OT-I cells at day 6 using gene set enrichment analysis (GSEA) with signatures derived from terminally exhausted or progenitor-exhausted SIINFEKL-reactive CD8^+^ TILs from B16-OVA tumors^8^. Data are representative of three independent experiments (**a-c**) with three to five samples per experimental group in each experiment. One-way ANOVA with Dunnett’s multiple comparison correction (**a-b**), Weighted Kolmogorov–Smirnov test with false discovery rate multiple comparison correction (**d**). Dots represent independent replicates (**a-c**), horizontal lines and error indicate mean ± s.e.m. (**a-b**). Source data

To determine the effect of BACH2_DE_ on the phenotype and function of CD8^+^ T cells, we first performed chronic stimulation assays in vitro. Splenic CD8^+^ T cells were stimulated, transduced with BACH2_OE_, BACH2_DE-10%_, BACH2_DE-5%_ or empty vectors, and maintained in media supplemented with interleukin (IL)-2 alone (acute stimulation) or IL-2 and anti-CD3 antibodies (chronic stimulation) replaced every 2 days (Fig. 3a)^34,35^. Chronic stimulation was sufficient to induce terminal exhaustion of a proportion of cultured cells, as indicated by co-induction of PD-1 and TIM-3 expression (Fig. 3b). Using this assay, we observed that both BACH2_OE_ and BACH2_DE_ caused a substantial reduction in the frequency of TIM-3^+^PD-1^+^ terminally exhausted cells after chronic stimulation (Fig. 3c). Both BACH2_OE_ and BACH2_DE_ also caused higher levels of CD62L and TCF1 expression relative to EV (Extended Data Fig. 3a,b). However, while BACH2_OE_ caused decreased cytokine expression relative to EV upon 4-h restimulation of acutely activated T cells, this was not observed in BACH2_DE-10%_-transduced or BACH2_DE-5%_-transduced cells (Fig. 3d). In addition, BACH2_OE_ cells were significantly smaller in size than EV cells (consistent with compromised levels of activation^20^), but this was not the case with BACH2_DE-10%_ or BACH2_DE-5%_ (Fig. 3e).

To investigate how BACH2_DE_ influences gene expression at the transcriptional level, we sorted transduced cells after 6 days of chronic stimulation and performed bulk RNA-seq. Principal component analysis highlighted substantial differences between EV, BACH2_OE_ and BACH2_DE_ groups, but a high degree of similarity among BACH2_DE-10%_ and BACH2_DE-5%_ (Extended Data Fig. 3c). In comparison with EV-transduced cells, BACH2_DE-10%_ and BACH2_DE-5%_ induced a set of transcriptional changes shared with BACH2_OE_ (clusters A, D and F), and a set of unique transcriptional changes (cluster C), whereas BACH2_OE_ produced a large set of unique transcriptional changes not shared with BACH2_DE_ (clusters B and E; Fig. 3f). Among shared profiles, clusters A and D contained genes upregulated upon both BACH2_OE_ and BACH2_DE_, including genes associated with T cell stemness such as *Tcf7* (encoding TCF1), *Slamf6* and *Id3*; and cluster F contained genes downregulated upon both BACH2_OE_ and BACH2_DE_, including the known BACH2 target-genes associated with terminal T cell differentiation *Havcr2* (encoding TIM-3) and *Prdm1* (encoding BLIMP-1)^20^. Among uniquely regulated profiles, cluster E contained genes uniquely downregulated by BACH2_OE_, and associated with effector differentiation, including *Ccr5*, *Gzma*, *Ccr2*, *Cxcr6*, *Id2* and *Gzmc*; cluster B contained genes uniquely upregulated by BACH2_OE_, including *Ccl22*, *Dnmt1*, *Kit*, *Socs3* and *Tnf*; cluster C contained genes uniquely upregulated by BACH2_DE_, including *Ly6a*, *Cd266* and *Il18*. Notably, many of these genes, including *Cxcr6*, *Ccr2* and *Havcr2*, contained known BACH2 binding sites in the vicinity of their transcriptional start sites (TSSs)^20^ (Fig. 3f,g). Moreover, gene-set enrichment analysis (GSEA) showed that both BACH2_OE_ and BACH2_DE_ cells exhibited a transcriptional signature more closely aligned with that of stem-like T cells, while EV-transduced cells bore higher resemblance to the signature of terminally differentiated T cells (Extended Data Fig. 3d). Collectively, these data suggest that low-dose expression of BACH2 yields a total BACH2 level that is comparable or slightly higher to endogenous BACH2 levels found in central memory T cells and promotes retention of stem-like characteristics without compromising effector functions.

### Low-dose BACH2 partially attenuates AP-1 binding to control highly AP-1-dependent genes

**Fig. 4:**
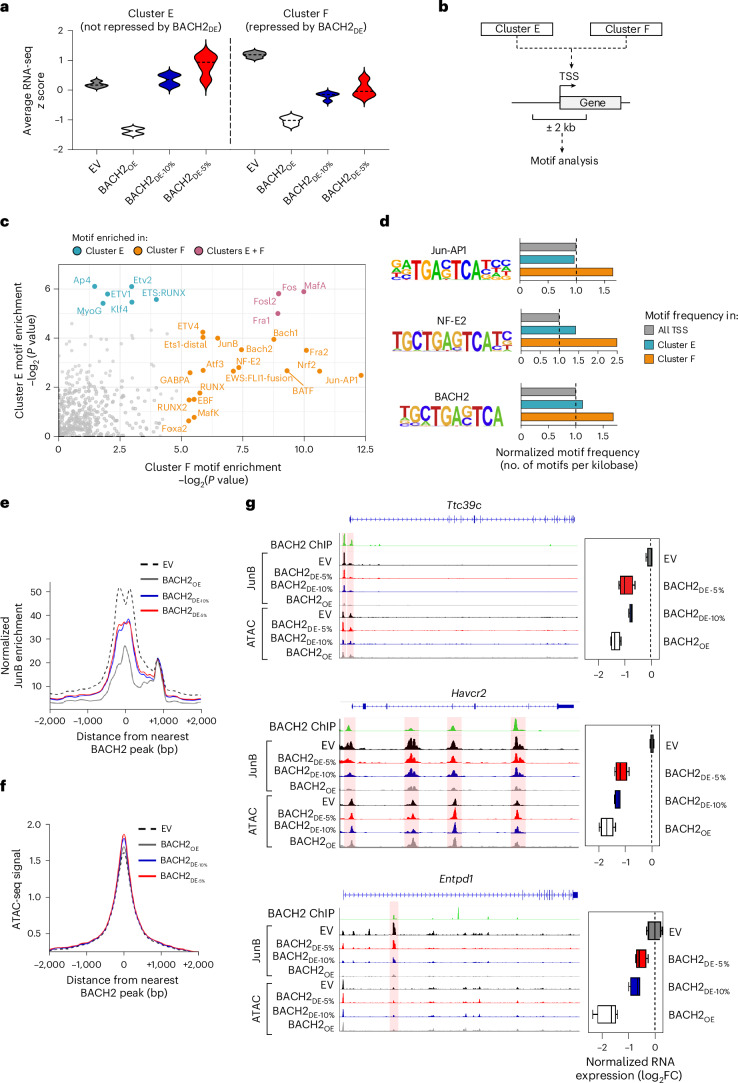
AP-1 motif enrichment and attenuated JunB binding are associated with sensitivity to repression by BACH2_DE_. **a**, Average normalized gene expression of genes within clusters E and F from RNA-seq experiment in Fig. 3f. **b**, Schema of TF motif enrichment analysis. The regions 2 kb upstream and downstream of the TSSs of all genes in clusters E and F were subjected to motif enrichment analysis. **c**, TF motifs enriched (–log_2_(*P* value) > 5) in the vicinity of the TSSs of clusters E and F. **d**, Frequency of selected TF motifs within the ±2-kb TSS region of genes in cluster E and cluster F normalized to their frequency around all known TSSs across the mouse genome. **e**,**f**, Average JunB binding as determined using CUT&RUN (**e**) and chromatin accessibility as determined using ATAC-seq (**f**) within chronically stimulated cells transduced with the indicated vectors relative to peak centers of annotated BACH2 binding sites^20^. **g**, Representative alignments showing JunB binding and chromatin accessibility at selected loci within chronically stimulated cells transduced with the indicated vectors (left). Normalized RNA-seq log_2_(FC) for corresponding genes from chronically stimulated cells transduced with the indicated vectors (*n* = 4 for all groups) are shown (right). Statistical values determined using hypergeometric distribution through HOMER (**c**). Samples used for RNA-seq and CUT&RUN constitute independent replicates. Box plots display the minimum and maximum values (whiskers), median (vertical line) and interquartile range (box) (**g**). In **g**, regions where BACH2 ChIP signal and JunB CUT&RUN signal align are highlighted with red shading. Source data

To understand the mechanistic basis for differential gene regulation by BACH2_OE_ versus BACH2_DE_, we analyzed DNA sequences located within ±2 kb of the TSSs of genes differentially repressed by BACH2_OE_ and BACH2_DE_. We focused on genes from clusters E and F identified in our RNA-seq analysis: cluster E genes were repressed only by BACH2_OE_, while cluster F genes were repressed by both BACH2_OE_ and BACH2_DE_ (Fig. 4a,b). Motif enrichment analysis revealed that cluster F promoters were significantly more enriched for bZIP binding sites containing the AP-1 consensus TRE palindromic sequence TGA(G/C)TCA, corresponding to motifs associated with TFs such as BATF, Fra2, JunB and Atf3 (Fig. 4c). Indeed, the frequency of TRE-containing bZIP motifs was consistently higher in cluster F promoters compared with both cluster E and genome-wide promoter regions (Fig. 4d). These findings suggest that genes susceptible to low-dose BACH2 regulation are distinguished by higher frequencies of AP-1 motifs in the vicinity of their TSSs, potentially indicating higher AP-1 dependency.

**Extended Data Fig. 4:**
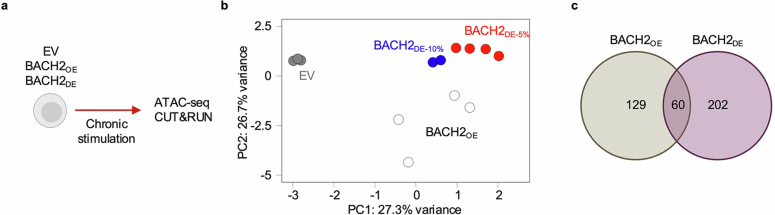
High and low expression of BACH2 drive changes in chromatin accessibility upon chronic stimulation. **a**, Experimental schema. CD8^+^ T cells were isolated, transduced with EV (*n* = 3), BACH2_OE_ (*n* = 4), BACH2_DE-10%_ (*n* = 2) and BACH2_DE-5%_ (*n* = 4) and subjected to chronic stimulation prior to ATAC-seq or CUT&RUN. **b**, ATAC-seq PCA plot of indicated sample groups. **c**, Number of differentially accessible chromatin regions (FC > 2, FDR < 0.1) in BACH2_OE_ and BACH2_DE_ relative to EV. Dots represent independent replicates (**b**).

Because BACH2 functions as an AP-1 repressor in CD8⁺ T cells, we asked whether AP occupancy at BACH2 binding sites is differentially regulated by BACH2_OE_ and BACH2_DE_^20^. To test this, we performed Cleavage Under Targets & Release Using Nuclease (CUT&RUN) for the AP-1 factor JunB and assay for transposase-accessible chromatin sequencing (ATAC-seq) for chromatin accessibility in chronically stimulated CD8^+^ T cells transduced with BACH2_OE_ and BACH2_DE_ vectors. While ATAC-seq analysis showed global changes in genome-wide chromatin accessibility consistent with the distinct differentiation states of BACH2_OE_ and BACH2_DE_ CD8^+^ T cells, these changes were not enriched at BACH2 binding sites. Nonetheless, JunB occupancy showed dose-dependent attenuation (Fig. 4e,f and Extended Data Fig. 4a–c). BACH2_OE_ caused near-complete loss of JunB binding at BACH2 sites, whereas BACH2_DE_ resulted in partial reduction in JunB binding frequency compared to cells transduced with an EV. This graded AP-1 displacement was evident at the regulatory elements of effector and exhaustion-associated genes including *Ttc39c*, *Havcr2* and *Entpd1*, with transcript levels showing corresponding dose-dependent changes (Fig. 4g).

**Extended Data Fig. 5:**
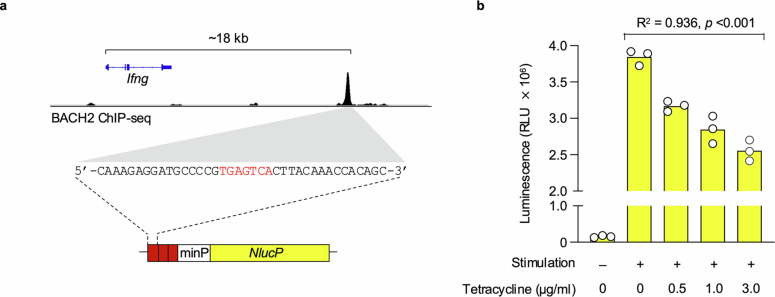
Inducible BACH2 expression shows dose-dependent AP-1 suppression. **a**, Schematic of the BACH2 reporter assay system. The DNA sequence ~18 kb downstream of the *Ifng* locus containing a TPA responsive element (TRE) bound by BACH2 was cloned in triplicate upstream of a minimal promoter (minP) driving NlucP luciferase expression^20,23^. This reporter construct was stably integrated into Jurkat cells along with a tetracycline-inducible BACH2 expression cassette, enabling dose-dependent analysis of BACH2-mediated transcriptional repression as reported in Vardaka et al. **b**, Dose-dependent repression of AP-1-driven luciferase activity by BACH2. Reporter cells were pre-treated with the indicated concentrations of tetracycline for 48 h to model continuous pre-existing BACH2 expression at distinct levels and then stimulated with PMA and ionomycin for 6 h to induce AP-1 activity. Luciferase signal shows inverse correlation with tetracycline concentration (R² = 0.936, *p* < 0.001), demonstrating dose-dependent repression of AP-1-driven transcription by BACH2. Log- linear regression (**b**). Dots represent independently cultured and treated replicates (**b**), bars and error indicate mean ± SEM (**b**). Source data

To confirm that BACH2 mediates dose-dependent repression of AP-1-driven gene expression under conditions modeling constitutive expression, we utilized a previously developed reporter assay for BACH2-mediated repression of AP-1-driven gene expression^23^. In this assay, a Jurkat cell line harbors a luciferase reporter driven by three tandem copies of regions containing AP-1 consensus TRE palindromic TGA(G/C)TCA sequences derived from the *Ifng* + 18k enhancer, along with a tetracycline-inducible BACH2 expression system (Extended Data Fig. 5a). Using 48-h pretreatment with varying doses of tetracycline to model distinct levels of continuous BACH2 expression, we observed dose-dependent repression of phorbol myristate acetate (PMA)-induced luciferase activity, with intermediate repression at low BACH2 doses (Extended Data Fig. 5b). These findings confirm that BACH2 functions as a dose-dependent regulator of AP-1-driven transcription.

Together, these analyses support a model whereby BACH2_DE_ achieves selective gene regulation through partial AP-1 displacement, with AP-1-dependent genes being preferentially sensitive to low-dose BACH2 regulation.

### Low-dose BACH2 enhances antitumor T cell responses

**Fig. 5:**
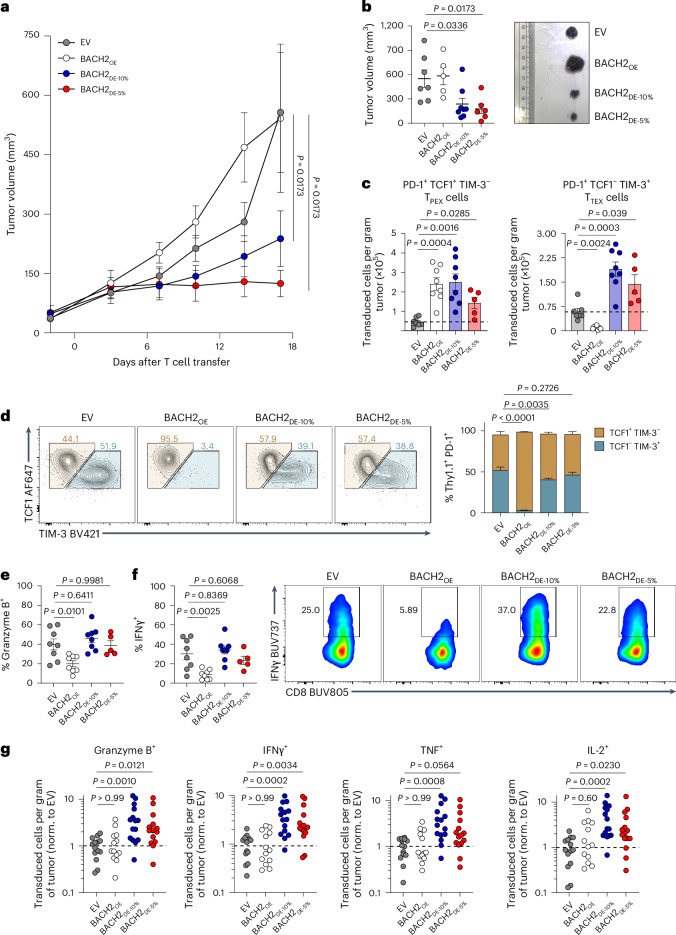
BACH2 dosing enhances antitumor T cell therapy responses. **a**, Tumor volume of B16-OVA-bearing mice following sublethal irradiation with 3.5 Gy and adoptive transfer of 0.5 × 10^6^ OT-I T cells transduced with EV (*n* = 7), BACH2_DE-5%_ (*n* = 4), BACH2_DE-5%_ (*n* = 8) and BACH2_OE_ (*n* = 5). Mice euthanized due to reasons unrelated to tumor size were excluded. **b**, Tumor volumes at days 15–17 from mice alive at the time of measurement after T cell transfer and representative images of B16-OVA tumors. Ruler scale is in cm. **c**, Number of tumor-infiltrating transduced OT-I T cells per gram of tumor from **a** for indicated T cell phenotypes and sample groups. The horizontal dashed line represents the EV average. **d**, Quantification of TCF1 and TIM-3 frequency of expression in tumor-infiltrating transduced PD-1^+^ OT-I T cells and representative flow cytometry plots. Significance represents difference in frequency of TCF1^+^TIM-3^−^ cells with that of the EV population. **e**,**f**, Frequency of granzyme B^+^ (**e**) and IFNγ^+^ (**f**) transduced intratumoral OT-I T cells from EV (*n* = 8), BACH2_DE-5%_ (*n* = 5), BACH2_DE-5%_ (*n* = 8) and BACH2_OE_ (*n* = 8) following ex vivo 4-h restimulation and representative flow cytometry plots (**f**). **g**, Quantification of the absolute number of tumor-infiltrating transduced OT-I T cells from EV (*n* = 14), BACH2_DE-5%_ (*n* = 15), BACH2_DE-5%_ (*n* = 15) and BACH2_OE_ (*n* = 13) per gram of tumor expressing the indicated effector molecule normalized to EV. Data are representative of three independent experiments. Data in **g** represent pulled independent replicates from two independent experiments. One-way ANOVA with Dunnett’s multiple-comparison correction (**a**–**f**); Kruskal–Wallis test with Dunn’s multiple-comparison correction (**g**). Tumor curves represent the mean of independent replicates ± s.e.m. (**a**), dots represent independent replicates (**b**, **c** and **e**–**g**), and bars and errors indicate the mean ± s.e.m. (**b**–**g**). Source data

**Extended Data Fig. 6:**
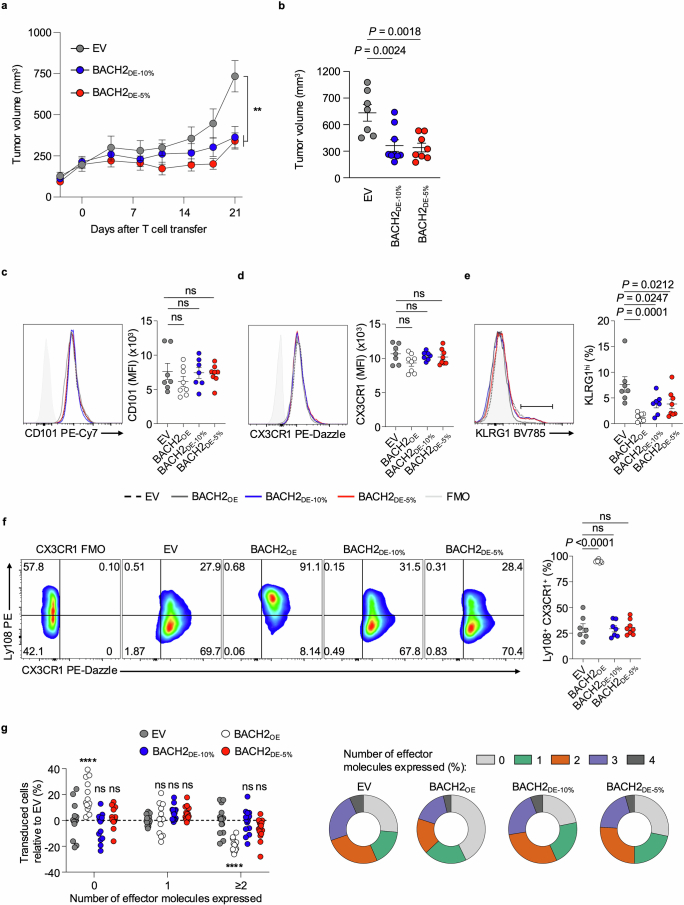
BACH2 dosing enhances anti-tumor T cell therapy responses in MC38-OVA tumor-bearing mice. **a**, Tumor volume of MC38-OVA-bearing mice following sublethal irradiation with 2.5 Gy and adoptive transfer of 0.5×10^6^ OT-I T cells transduced with EV (*n* = 7), BACH2_DE-10%_ (*n* = 9) and BACH2_DE-5%_ (*n* = 8) vectors. **b**, Tumor volumes from (a) at days 21 after T cell transfer. **c-f**, MFI of CD101 (**c**), CX3CR1 (**d**) and frequency of KLRG1^hi^ cells (**e**) and Slamf6^+^ CX3CR1^+^ in intratumoral OT-I T cells transduced with EV (*n* = 7), BACH2_DE-10%_ (*n* = 7) and BACH2_DE-5%_ (*n* = 8) and BACH2_OE_ (*n* = 8) vectors and representative flow cytometry histograms. Fluorescence minus one (FMO) controls for the respective markers is shown. **g**, Absolute difference in the frequency of tumor-infiltrating transduced OT-I T cells from EV (*n* = 14), BACH2_DE-5%_ (*n* = 15), BACH2_DE-5%_ (*n* = 15) and BACH2_OE_ (*n* = 13) expressing the indicated number of effector molecules (IFN-g, TNF, granzyme B, IL-2) upon 4-hour *ex vivo* anti-CD3 stimulation normalized to EV. Pie charts represent the average proportion of cells from each condition (co-) expressing the indicated number of effector molecules. Data in (**e-f**) represent pooled independent replicates from two independent experiments. ns, non-significant (*P* > 0.05); *, *P* < 0.05; **, *P* < 0.01; ****, *P* < 0.0001. One-way ANOVA with Dunnett’s multiple comparison correction (**b**, **g**). Tumor curve represents average of independent replicates ± s.e.m. (**a**), dots represent independent replicates (**b**-**g**), horizontal lines and error indicate mean ± s.e.m. (**b-g**). Source data

The ability of BACH2 dosing to enable retention of a stem-like phenotype without compromising effector functions led us to ask whether this approach can be utilized to enhance adoptive T cell therapy responses in vivo. B16-OVA tumor-bearing mice were intravenously administered with OT-I T cells transduced with empty, BACH2_OE_ or BACH2_DE_ vectors. While BACH2_OE_ was unable to enhance the antitumor efficacy of adoptively transferred OT-I T cells compared with EV-transduced cells, BACH2_DE-10%_-transduced and BACH2_DE-5%_-transduced OT-I cells mediated substantially enhanced antitumor responses (Fig. 5a,b). Similar results were obtained with an OVA-expressing MC38 colorectal carcinoma T cell therapy model (MC38-OVA; Extended Data Fig. 6a,b).

Notably, while BACH2_OE_ led to an increase in the absolute number of T_PEX_ cells but a decrease in T_TEX_ cells relative to EV control, both BACH2_DE-10%_ and BACH2_DE-5%_ resulted in increased numbers of both T cell subsets (Fig. 5c). Consequently, phenotypic marker analysis revealed that BACH2_OE_ resulted in a near-complete loss of terminally differentiated TCF1^−^TIM-3^+^ T_TEX_ cells, while the overall frequency of stem-like and terminally differentiated cells remained minimally altered among BACH2_DE_-transduced cells (Fig. 5d and Extended Data Fig. 6c–e). Evaluation of effector cytokine production upon 4-h ex vivo restimulation revealed that BACH2_OE_-transduced cells displayed a significantly lower frequency of cells expressing effector molecules (IFNγ, TNF, granzyme B, IL-2), while this remained unchanged between BACH2_DE_ and EV control (Fig. 5e,f and Extended Data Fig. 6f). Consistent with the observed expansion of both T_PEX_ and T_TEX_ subsets and preserved effector functions, mice receiving BACH2_DE_-transduced cells displayed a significantly increased number of cytokine-producing cells per gram of tumor (Fig. 5g). Collectively, these data suggest that BACH2_DE_ enhances the antitumor efficacy of CD8^+^ T cells by promoting persistence while allowing acquisition of effector functions.

### Low-dose BACH2 induces a hybrid transcriptional state among Slamf6^−^ cells

**Fig. 6:**
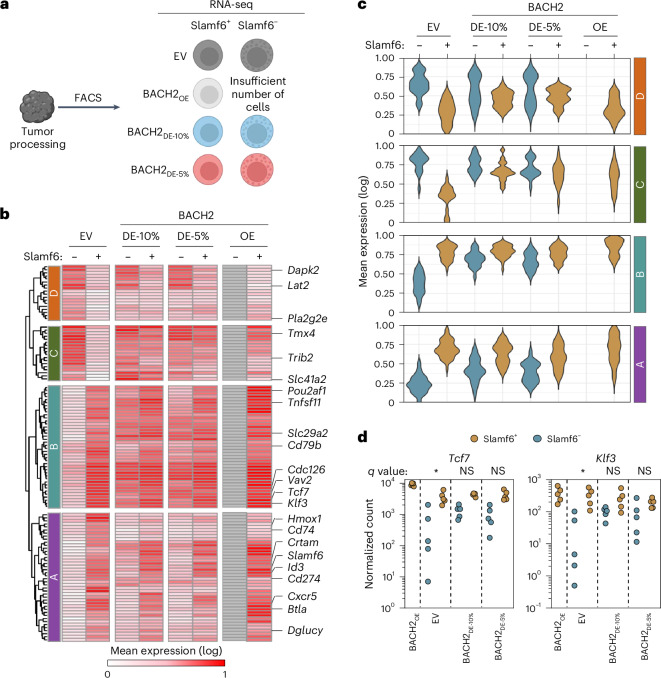
BACH2 dosing drives hybrid stem and effector state among Slamf6⁻ cells and limited changes among Slamf6⁺ cells. **a**, Experimental schema. Tumor-infiltrating transduced OT-I T cells were isolated from B16-OVA tumor-bearing mice 18 days after T cell transfer and sorted via fluorescence-activated cell sorting (FACS) into Slamf6^+^ and Slamf6^−^ fractions for analysis by bulk RNA-seq. Insufficient cell numbers were recovered from Slamf6^−^ cells in mice receiving BACH2_OE_-transduced cells. **b**, Heat map displaying average log_2_ gene expression normalized to row maxima within indicated populations. Genes displayed correspond to all DEGs (*q* < 0.05, log_2_(FC) > 1) between Slamf6^+^ and Slamf6^−^ in EV and BACH2_DE_. **c**, Violin plots displaying the distribution of gene expression values of genes within each of the clusters from **b** within indicated populations. **d**, Normalized expression counts of *Tcf7* and *Klf3* from indicated sample groups. Significance shown represents *q* values from expression comparisons between Slamf6^+^ and Slamf6^−^ in each condition. Samples used for RNA-seq are independent replicates (*n* = 5 for all groups). Dots represent independent replicates (**d**). NS (*P* > 0.05); *P < 0.05. Statistical significance determined via DESeq2 using a Wald test with Benjamini–Hochberg multiple-comparison correction (**d**). **a** was created with BioRender.com. Source data

To better understand how constitutive low-dose BACH2 expression affects CD8^+^ tumor-reactive T cells in distinct differentiation states, we sorted BACH2_OE_-transduced or BACH2_DE_-transduced OT-I CD8^+^ T cells from B16-OVA tumors based on Slamf6 expression, which marks progenitor-exhausted cells, and performed RNA-seq analysis (Fig. 6a). As previously observed, BACH2_OE_ limited differentiation of T_TEX_ cells, resulting in insufficient Slamf6^−^ cells for analysis.

**Extended Data Fig. 7:**
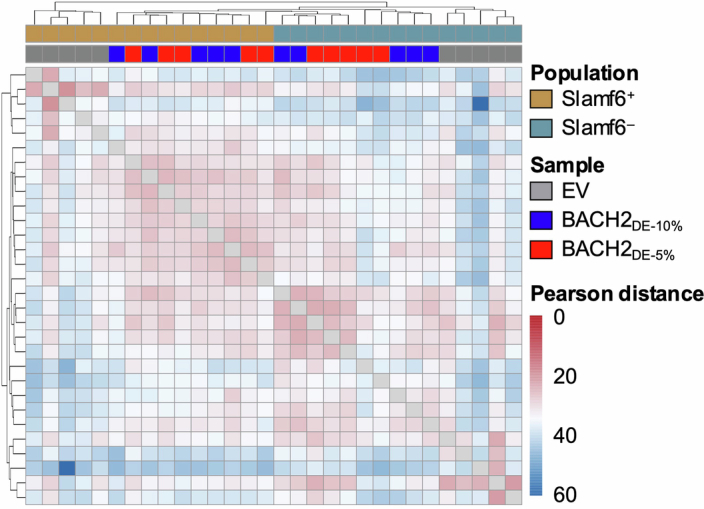
Low-dose expression of BACH2 induces changes in a Slamf6–-specific manner. Pearson similarity matrix illustrating Slamf6^+^ and Slamf6^−^ populations from intratumoral OT-I T cells transduced from the indicated conditions (*n* = 5 for all groups). Samples represent independent replicates.

Hierarchical clustering revealed four distinct gene expression patterns showing how BACH2_DE_ differentially affects Slamf6^+^ and Slamf6^−^ populations (Fig. 6b,c). Cluster A genes (*Cxcr5, Id3, Slamf6*) were genes predominantly expressed by Slamf6^+^ cells in both EV and BACH2_DE_ conditions, with minimal expression in Slamf6^−^ cells even after BACH2_DE_ treatment. This suggests that BACH2_DE_ leaves key aspects of the transcriptional program among Slamf6^−^ cells intact rather than imposing the full T_PEX_ transcriptional program upon them. Similarly, cluster D contained genes characteristic of Slamf6^−^ cells regardless of BACH2_DE_ transduction. The most notable transcriptional changes occurred in clusters B and C. Cluster B contained genes including T_PEX_-associated transcription factors (*Tcf7, Klf3*) whose expression is normally restricted to Slamf6^+^ cells but that were induced in Slamf6^−^ cells by BACH2_DE_, suggesting that BACH2_DE_ induces a limited set of stem-like transcriptional characteristics among Slamf6^−^ cells while maintaining their core differentiated transcriptional program (Fig. 6d). Interestingly, cluster C contained genes characteristic of Slamf6^−^ cells (*Hmox1, Cd74, Crtam*), which became expressed by Slamf6^+^ cells upon BACH2_DE_ expression, indicating that BACH2 dosing induces transcriptional changes in both populations, although the effects are most pronounced in Slamf6^−^ cells. Consistent with these changes, similarity matrix analysis showed that BACH2_DE_ Slamf6^+^ and Slamf6^−^ populations cluster more closely than their EV counterparts (Extended Data Fig. 7). Together, these data reveal that BACH2 dosing drives transcriptional changes to both Slamf6^+^ and Slamf6^−^ cells, with the most substantial changes to the Slamf6^−^ subset in which it drives a hybrid transcriptional state promoting acquisition of a limited set of stem-like transcriptional characteristics while also enabling them to retain their more differentiated identity.

**Fig. 7:**
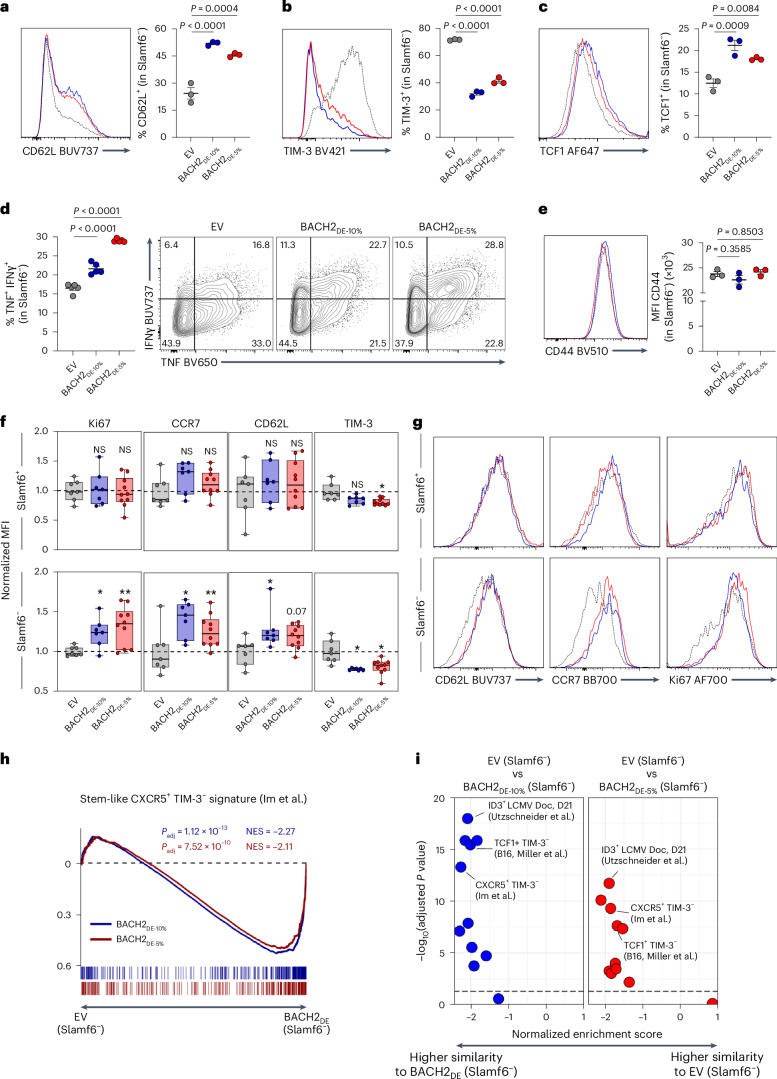
BACH2 dosing enables retention of stem-like characteristics in Slamf6^−^ cells without compromising effector functions. **a**–**c**, Frequency of CD62L^+^(**a**), TIM-3^+^ (**b**) and TCF1^+^ (**c**) cells within the Slamf6^**−**^ population transduced with the indicated vectors (*n* = 3 for all groups) after chronic stimulation and representative flow cytometry histograms. **d**, Frequency of IFNγ^+^ TNF^+^ within the Slamf6^**−**^ population transduced with the indicated vectors (*n* = 5 for all groups) upon 4 h of PMA and ionomycin restimulation in the presence of brefeldin A and monensin following chronic stimulation and representative flow cytometry plots. **e**, MFI of CD44 within the Slamf6^**−**^ population transduced with the indicated vectors (*n* = 3 for all groups) upon chronic stimulation and representative flow cytometry histograms. **f**,**g**, Comparison of the MFI (normalized to EV) of indicated markers between Slamf6^+^ (top) and Slamf6^−^ (bottom) in tumor-infiltrating adoptively transferred OT-I T cells transduced with from EV (*n* = 7), BACH2_DE-5%_ (*n* = 10) and BACH2_DE-10%_ (*n* = 7) (**f**), and representative flow cytometry histograms (**g**). **h**,**i**, Representative example of GSEA analysis comparing Slamf6^−^ EV and Slamf6^−^ BACH2_DE-10%_-transduced (blue) or BACH2_DE-5%_-transduced (red) OT-I T cells sorted from B16-OVA tumors using publicly available T_PEX_ signatures as reference gene sets (**h**) and normalized enrichment scores using publicly available T_PEX_ gene sets (**i**). The horizontal dashed line in i represents an adjusted *P* value of 0.05. Data are representative of two independent experiments (**a**–**g**). NS (*P* > 0.05); *P < 0.05; **P < 0.01. One-way ANOVA with Dunnett’s multiple-comparison correction (**a**–**f**). Weighted Kolmogorov–Smirnov test with false discovery rate multiple-comparison correction (**h** and **i**). Dots represent independent replicates (**a**–**f**), bars and errors indicate the mean ± s.e.m. (**a**–**e**), and box plots display the minimum and maximum value (whiskers), median (vertical line) and interquartile range (box; **f**). Source data

**Extended Data Fig. 8:**
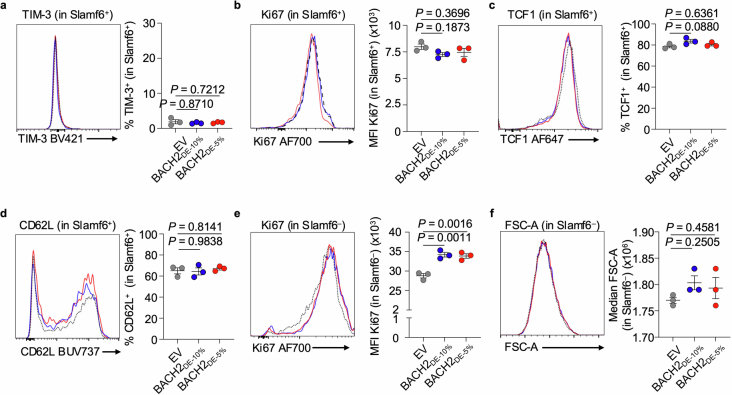
BACH2 dosing does not induce phenotypic changes in Slamf6+ cells in vitro. **a-b**, MFI of Ki67 (**a**) and median FSC-A (**b**) within the Slamf6^−^ population transduced with the indicated vectors (*n* = 3 for all groups) upon chronic stimulation upon chronic stimulation and representative flow cytometry histograms. **c-f**, Frequency of TCF1 (**c**), CD62L (**d**), and TIM-3 (**e**) or MFI of Ki67 (**f**), within rested Slamf6^+^ population transduced with the indicated vectors. Data are representative of two independent experiments. One-way ANOVA with Dunnett’s multiple comparison correction. Dots represent independent replicates, horizontal lines and error indicate mean ± SEM. Source data

During physiological CD8^+^ T cell differentiation, stemness and effector function exist in an inverse relationship, with quiescence factors maintaining stem-like properties through active suppression of effector programs^36–38^. Our transcriptional analyses suggested that BACH2 dosing may disrupt this relationship, creating a hybrid differentiation state among Slamf6^−^ cells combining the transcriptional characteristics of stem-like and effector cells. To test whether this corresponds to a hybrid phenotype, we performed phenotypic and functional analyses within in vitro and in vivo settings. We first subjected OT-I T cells transduced with EV or BACH2_DE_ vectors to either acute or chronic stimulation conditions, allowing generation of Slamf6^+^ and Slamf6^−^ cells, respectively. Among chronically stimulated CD8^+^ T cells, BACH2_DE_ selectively increased the frequency of CD62L^+^ and TCF1^+^ cells within the Slamf6^−^ subset while reducing TIM-3 expression relative to Slamf6^−^ EV cells, consistent with BACH2_DE_ driving retention of stem-like features among Slamf6^−^ cells (Fig. 7a–c). On the other hand, acutely stimulated Slamf6^+^ cells transduced with BACH2_DE_ vectors showed no significant changes in TCF1, CD62L, TIM-3 or Ki67 expression compared to Slamf6^+^ EV (Extended Data Fig. 8a–d), consistent with the less substantial transcriptional differences driven by BACH2_DE_ within this subset. However, despite acquiring features associated with less differentiated stem-like CD8^+^ T cells, BACH2_DE_-transduced Slamf6^−^ cells exhibited effector characteristics, including increased production of IFNγ and TNF upon restimulation compared to EV-transduced cells, increased Ki67, maintained cell size and similar CD44 expression (Fig. 7d,e and Extended Data Fig. 8e,f).

Consistent with in vitro observations, intratumoral Slamf6^−^ cells resulting from the adoptive transfer of BACH2_DE_-transduced OT-I T cells into B16-OVA-bearing mice displayed elevated CD62L, CCR7 and Ki67 expression compared to EV controls, whereas BACH2_DE_ and BACH2_OE_-transduced Slamf6^+^ cells possessed largely similar phenotypic characteristics, except for mildly reduced TIM-3 MFI (Fig. 7f,g). This was associated with significant enrichment of multiple T_PEX_-associated gene sets among BACH2_DE_-transduced Slamf6^−^ cells compared to EV-transduced Slamf6^−^ cells (Fig. 7h,i)^8,39–41^.

Together, these data demonstrate that BACH2 dosing drives a nonphysiological hybrid differentiation state among Slamf6^−^ cells, featuring retention of a set of transcriptional and phenotypic characteristics of T_PEX_ cells, while leaving the core effector differentiation program intact. BACH2 dosing also drove a milder set of transcriptional changes among Slamf6^+^ cells, which like Slamf6^−^ cells accumulated to higher frequencies within tumors upon BACH2_DE_ but was associated with more minimal changes in the phenotype of cells.

### Low-dose constitutively active FOXO1 enhances antitumor T cell responses

**Fig. 8:**
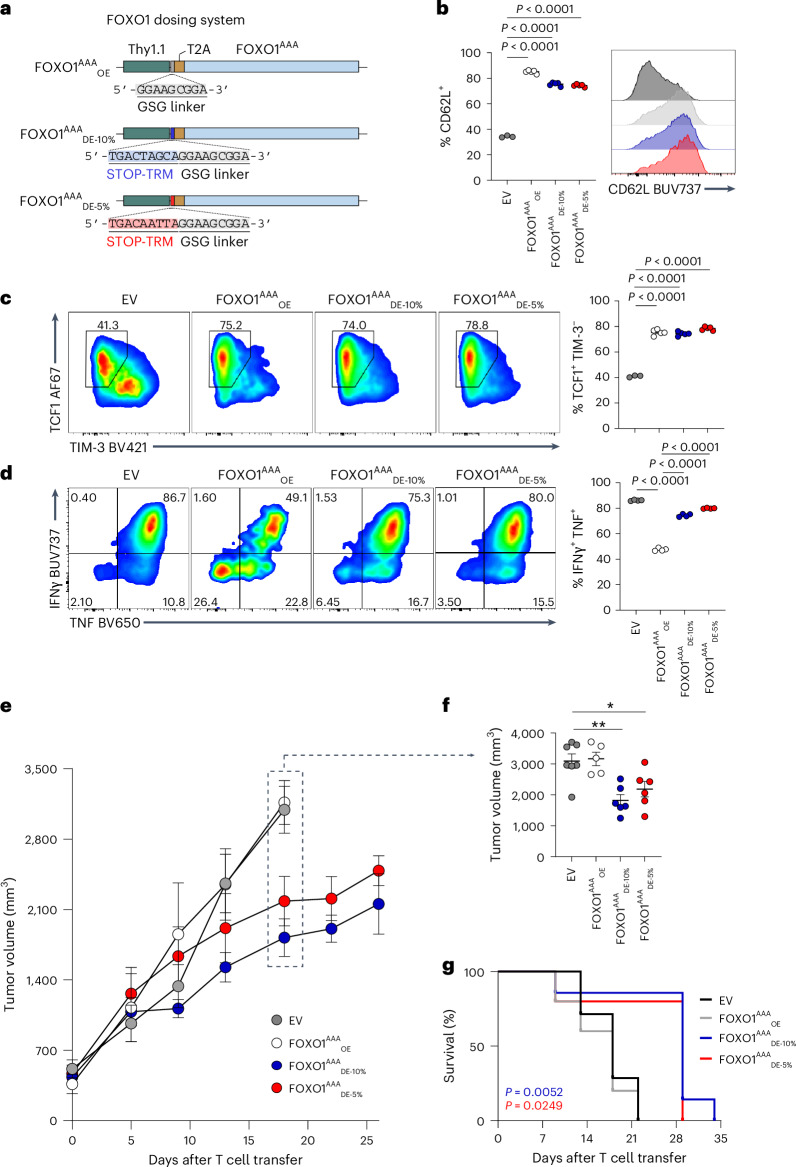
Low-dose FOXO1^AAA^ promotes stemness without compromising effector functions and enhances antitumor responses. **a**, Design of FOXO1^AAA^_OE_ and FOXO1^AAA^_DE_ vectors. **b**–**d** Frequency of CD62L^+^ chronically stimulated cells (**b**), TCF1^+^TIM-3^−^ chronically stimulated cells (**c**) or IFNγ^+^ TNF^+^ acutely stimulated cells following 4 h of anti-CD3 restimulation in the presence of brefeldin A and monensin (**d**) from OT-I T cells transduced with EV (*n* = 3 no restimulation, *n* = 5 restimulation), FOXO1^AAA^_DE-5%_ (*n* = 5), FOXO1^AAA^_DE-10%_ (*n* = 5) and BACH2_OE_ (*n* = 5) vectors at day 4 and representative flow cytometry histograms/plots. **e**,**f**, Tumor volumes of B16-OVA-bearing mice following sublethal irradiation with 3.5 Gy and adoptive transfer of 0.5 × 10^6^ OT-I T cells transduced with EV (*n* = 7), FOXO1^AAA^_DE-5%_ (*n* = 6), FOXO1^AAA^_DE-10%_ (*n* = 6) and BACH2_OE_ (*n* = 5) vectors (**e**) and tumor volumes at day 18 after T cell transfer (**f**). For mice that were euthanized, the final tumor volume was carried forward and included in the average. Tumor volumes are shown up to the time point when >20% of mice remained alive. **g**, Kaplan–Meier survival curve of B16-OVA-bearing mice shown in **e**. Significant differences represent differences between EV and either FOXO1^AAA^_DE-10%_ (blue) or FOXO1^AAA^_DE-5%_ (red). Data are representative of two independent experiments. *P < 0.05; **P < 0.01. One-way ANOVA with Dunnett’s multiple-comparison correction (**b**–**d** and **f**), Kaplan–Meier log-rank Mantel–Cox test (**g**). Dots represent independent replicates (**b**–**d** and **f**), and horizontal lines and errors indicate the mean ± s.e.m. Tumor curve represents the average of independent replicates ± s.e.m. (**e**). Source data

To extend this work beyond BACH2 and to test whether dose optimization is a generalizable requirement for effective deployment of quiescence factors, we tested the relevance of dose optimization with the quiescence factor FOXO1 (refs. ^42–45^). Similarly to BACH2, FOXO1 is required for maintenance of memory and progenitor-exhausted CD8^+^ T cells, and is more highly expressed in naive CD8^+^ T cells than in central memory and effector memory subsets^42–44^. We cloned vectors expressing a constitutively active triple-alanine mutant of FOXO1 (FOXO1^AAA^)^46^, using the STOP-TRM system enabling either conventional high-dose overexpression or dosed expression of FOXO1^AAA^ (FOXO1^AAA^_OE_, FOXO1^AAA^_DE-10%_ and FOXO1^AAA^_DE-5%_; Fig. 8a). Using an in vitro chronic stimulation assay, we found that both high-dose and low-dose constitutive expression of FOXO1^AAA^ led to a comparable increase in the frequency of CD62L^+^ and TCF1^+^TIM-3^−^ cells, relative to EV-transduced cells (Fig. 8b,c). However, FOXO1^AAA^_OE_ caused a substantial impairment in the production of IFNγ and TNF after 4-h brief restimulation in vitro, while this was not the case for FOXO1^AAA^_DE-10%_ and FOXO1^AAA^_DE-5%_ (Fig. 8d).

We next treated B16-OVA-bearing mice with OT-I T cells transduced with empty, high-dose or low-dose FOXO1^AAA^ vectors. As previously observed with the BACH2 dosing vectors, both FOXO1^AAA^_DE-10%_ and FOXO1^AAA^_DE-5%_ groups mediated a significant improvement in antitumor responses relative to EV, but this was not the case with the FOXO1^AAA^_OE_ group (Fig. 8e,f). This also resulted in an overall improvement in survival resulting from transduction of adoptively transferred cells with FOXO1^AAA^_DE_ vectors (Fig. 8g). These data suggest that dose optimization is a generalizable requirement for effective deployment of quiescence factors such as BACH2 and FOXO1, with quantitative changes to payload expression resulting in qualitatively distinct changes to the phenotypic and functional output of cells.

## Discussion

CAR T cell therapies are revolutionizing treatment of hematological malignancies, but major barriers exist to effective treatment of solid cancers^47,48^. Prior attempts to enhance T cell persistence have largely relied on enforcing constitutively activated cellular states, including through overexpression of proto-oncogenes such as JUN and MYB, constitutively active STAT5 variants, and the CARD11–PIK3R3 oncogenic fusion protein^5,16,18,17^. While these strategies have shown preclinical efficacy by maintaining cells in persistently activated states, the need to constitutively overexpress known proto-oncogenes raises concerns about their potential to drive excessive activation or therapy-derived lymphomas^25^.

In the present study, we show that dose-optimized expression of the quiescence factor BACH2 enhances the persistence and antitumor efficacy of adoptive T cell therapy. Rather than enforcing persistent activation, BACH2 restrains effector programs, promoting a state of regulated quiescence that more closely resembles physiological T cell maintenance. The known role of BACH2 as a tumor suppressor in the context of CAR T cell-derived lymphomas raises the potential that this approach may also protect against therapy-induced lymphomagenesis rather than potentiating it, representing a potentially safer strategy for enhancing T cell persistence in cellular immunotherapy^24,25^. Moreover, the quiescence factor function of BACH2 may result in ‘slower release’ of effector cytokines, potentially reducing the possibility of cytokine release syndrome. In addition, in this study, we utilized the OT-I TCR model to investigate the effects of dosed BACH2 expression on antitumor efficacy. Notably, CARs differ from the TCR system in their tendency to drive ligand-independent tonic signaling, which can lead to T cell dysfunction and terminal differentiation^49^. Given its role in limiting T cell differentiation, it is conceivable that BACH2 dosing may be able to shield CAR T cells from ligand-independent tonic signaling, as well as from the effects of antigen-driven chronic stimulation.

BACH2 dosing had a greater impact on the transcriptional and phenotypic characteristics of Slamf6^−^ cells than Slamf6^+^ cells. Our data showed that Slamf6^+^ cells express higher levels of endogenous *Bach2* than Slamf6^−^ cells on a per-cell basis, suggesting that constitutive low-dose BACH2 expression represents a more substantial increase in total BACH2 levels for Slamf6^−^ cells than for Slamf6^+^ cells. Indeed, BACH2_DE_ resulted in BACH2 protein levels comparable to those in central memory T cells, which express intermediate levels of endogenous BACH2. This differential impact on Slamf6^−^ cells is consistent with a model whereby BACH2 dosing establishes a lower limit of BACH2 expression that has its greatest effect on terminally differentiated cells that would otherwise experience lower BACH2 levels. Ultimately, this results in the acquisition of a nonphysiological hybrid differentiation state by Slamf6^−^ cells.

We tested whether our findings relating to BACH2 are generalizable to other quiescence factors. Examining a constitutively active FOXO1 variant (FOXO1^AAA^), we found that, similarly to BACH2, dose optimization was critical for therapeutic efficacy. These findings align with recent reports from two independent laboratories showing that overexpression of wild-type FOXO1 improves CAR T cell antitumor responses^50,51^. Notably, both studies found that wild-type FOXO1, but not FOXO1^AAA^, was able to enhance antitumor responses in CAR T cell models in vivo. Our results provide a mechanistic explanation for this observation: the heightened activity of FOXO1^AAA^ overexpression restricts effector functions similarly to our FOXO1^AAA^_OE_, while wild-type FOXO1—whose activity is attenuated through robust posttranslational regulation—may achieve activity levels more comparable to dose-optimized FOXO1^AAA^_DE_. Given the ability of FOXO1 to suppress effector functions is dependent upon BACH2 (ref. ^43^), BACH2 may serve as a downstream mediator in CAR T cells overexpressing FOXO1, warranting further investigation of this regulatory axis.

This study demonstrates that precise control of quiescence factor expression levels is critical for programming optimal T cell responses in the context of cellular immunotherapy. More broadly, these findings reveal that quantitative modulation of the expression of genetic payloads can yield qualitatively distinct cellular outcomes, with important implications for gene engineering approaches, and that other promising genetic payloads may have been overlooked in high-throughput screens not because of inherent inefficacy but suboptimal expression levels^52^. Future cellular engineering efforts should incorporate systematic gene ‘dose–response’ analyses during payload development, potentially revealing therapeutic windows where gene payloads can safely enhance cell and gene therapies.

## Methods

### Mice

OT-I and *Ptprc*^a^ (CD45.1) congenic mice from a C57BL/6 background were obtained from the Jackson Laboratory^26^. *Bach2*^tdRFP^ mice were generated as previously described^31^. BACH2^FLAG^ mice were generated as previously described^33^. Wild-type C57BL/6 mice were purchased from Charles River Laboratories. Experiments were performed with 8- to 12-week-old animals using age- and sex-matched experimental groups. Mice were housed at the University of Cambridge University Biomedical Services (UBS) Gurdon Institute Facility under standard dark–light cycles, and temperature- and humidity-controlled conditions. Experiments were conducted in accordance with UK Home Office guidelines and were approved by the University of Cambridge Animal Welfare and Ethics Review Board. No mice in this study exceeded the maximum tumor burden of 15-mm average diameter specified in the UK Home Office project license relevant for this work. Genotyping was performed by Transnetyx.

### Cell lines and reagents

The B16-F10 murine melanoma cell line was purchased from the American Type Culture Collection. The MC38-OVA cell line was purchased from Vitro Biotech. The B78ChOVA-mCherry (B16-OVA) murine melanoma cell line was kindly provided by M. Krummel. The BACH2-inducible reporter Jurkat cell line was generated as previously described^23^. Platinum-E retroviral ecotropic packaging cells (Plat-E) were purchased from Cell Biolabs. Cell lines were passaged in DMEM (Gibco) supplemented with 10% heat-inactivated fetal bovine serum (Sigma-Aldrich), 1 mM sodium pyruvate (Gibco), 0.1 mM non-essential amino acids (Gibco), 2 mM glutamine (Gibco) and 100 U ml^−1^ streptomycin and penicillin (Gibco). Murine-reactive anti-CD3 (clone 145-2C11) and anti-CD28 (clone 37.51) antibodies were purchased from BioLegend. Recombinant human IL-2 (rhIL-2) was purchased from PeproTech and stored at −80 °C until use.

### Processing of tumor, spleen and lymph nodes

Spleens and lymph nodes were mechanically dissociated through 40-μm cell strainers. Red blood cells were lysed using ACK Lysing Buffer (Gibco). Tumors were digested in DMEM with 20 μg ml^−1^ DNase I (Roche) and 1 mg ml^−1^ collagenase (Sigma-Aldrich) for 30 min at 37 °C. Digested tumors were mechanically dissociated through 40-μm cell strainers and washed twice with PBS. TIL enrichment was performed using Lympholyte-M solution (Cedarlane Labs) according to the manufacturer’s instructions. For assessing cytokine production, single-cell suspensions were resuspended in media containing 20 ng ml^−1^ PMA (Sigma) and 1 µg ml^−1^ ionomycin (Sigma), or 5 µg ml^−1^ anti-CD3, together with 5 µg ml^−1^ brefeldin A (Sigma) and 5 µg ml^−1^ monensin (Sigma) for 4 h.

### Generation of retrovirus for mouse T cell transduction

Plasmids encoding murine stem cell virus-based vectors for expression of BACH2 (CCDS51135.1) or FOXO1^AAA^ with a 3xFLAG tag at the N terminus were purchased from VectorBuilder. Vectors contained an ORF flanked by murine stem cell virus long terminal repeat sequences, containing a Thy1.1 coding sequence, a STOP-TRM motif, a T2A self-cleavage sequence and the coding sequence of the gene of interest. Three alanine mutations (p.Thr24Ala, p.Ser253Ala and p.Ser316Ala) were introduced in the FOXO1 (CCDS17343.1) ORF for generating FOXO1^AAA^ (ref. ^46^). At 70–80% confluency in a T175 flask, Plat-E cells were co-transfected with 6.3 μg pCL-Eco retroviral packaging plasmid (Addgene, 12371) and 28.5 μg retroviral vector plasmid DNA of interest in 3.17 ml OptiMEM medium (Gibco) and 95 μl TransIT-293 transfection reagent (Mirus Bio). Transfected Plat-E cells were cultured at 37 °C 5% CO_2_ and viral supernatant harvested at 48 h and 72 h after transfection. Viral supernatant was centrifuged at 400*g* for 5 min to remove cellular debris and stored at −80 °C until use.

### Primary mouse T cell transduction

CD45.1^+^ OT-I splenocytes were activated for 24 h in complete RPMI media (RCM; Gibco) containing 100 IU ml^−1^ rhIL-2, 10 μg ml^−1^ anti-CD3 and 5 μg ml^−1^ anti-CD28 antibodies. Activated T cells were resuspended at 1 × 10^6^ cells per ml in viral supernatant containing 100 IU ml^−1^ rhIL-2 and 8 μg ml^−1^ polybrene transfection reagent (Merck). Cell suspensions were plated on non-tissue culture-treated plates and centrifuged at 2,000*g* 32 °C for 2 h with minimal acceleration and no brake. Following centrifugation, cell suspensions were cultured for 4 h, then washed and maintained at 1.25 × 10^6^ cells per ml in RCM containing 100 IU ml^−1^ rhIL-2 until use. Transduction efficiency was evaluated by flow cytometry 48 h following transduction.

### Flow cytometry and cell sorting

Single-cell suspensions were blocked with anti-mouse CD16/CD32 Fc block (BioXCell, 2.4G2) followed by live and dead cell discrimination with Fixable Viability Dye eFluor 780 (Thermo Fisher Scientific). Surface staining was performed for 30 min away from light at 4 °C. Intracellular staining of transcription factors and cytokines was performed overnight following fixation and permeabilization using the eBioscience Foxp3/Transcription Factor Staining Buffer Kit (Invitrogen) and BD Cytofix/Cytoperm Fixation/Permeabilization Kit (BD Biosciences), respectively^53^. Cell counts were obtained using 123count eBeads (Invitrogen). Samples were acquired using Cytek Aurora cytometers and data analyzed using FlowJo v10 (Tree Star). For FACS, single-cell suspensions were filtered resuspended in complete RPMI 1640 media before sorting. Cells were sorted into RPMI 1640 media supplemented with 50% FCS (Sigma) and kept cold throughout until subsequent use. Cell sorting was performed using BD Aria or MoFlo Astrios (Beckman Coulter) cell sorters. UMAP plots were generated using clustering unsupervised methods for high-dimensional cytometry data (CRUSTY)^54^.

### Adoptive cell transfer

C57BL/6 mice were injected subcutaneously into the flank with 1.25 × 10^5^ B16-OVA cells or 3 × 10^5^ MC38-OVA cells 12–14 days before T cell transfer. Once established, tumor-bearing mice were selected and randomized into experimental groups. Tumor area was measured every 3–4 days thereafter using electronic calipers and volume calculated as length × width^2^. Mice received 2.5 Gy (MC38-OVA-bearing mice) or 3.5 Gy (B16-OVA-bearing mice) total body X-ray irradiation 1 day before adoptive transfer. Transduced OT-I cells (5 × 10^5^) were intravenously injected into tumor-bearing mice. For analysis of tumor-infiltrating cells, mice were culled 17–21 days following T cell transfer. Staff performing intravenous injections and tumor measurements were blinded to the experimental groups.

### Assessment of endogenous BACH2 expression

B16-F10 cells (1.25 × 10^5^) were subcutaneously injected into the flanks of *Bach2*^tdRFP/+^ or *Bach2*^+/+^ (control) mice. Tumor and spleen samples were harvested after 16 days, processed as previously described and analyzed by flow cytometry for expression of phenotypic surface markers and tdRFP expression.

### Luciferase assay

The luciferase assay was performed as previously described^23^. Briefly, inducible BACH2 reporter Jurkat cells were pretreated with tetracycline (or vehicle) for 18 h (T8032, Sigma-Aldrich). Subsequently, cells were stimulated for 6 additional hours with PMA (25 ng ml^−1^) and ionomycin (1.25 µg ml^−1^) in the presence of tetracycline (or vehicle) before measuring luciferase signal using a PHERAstar FSX spectrophotometer (BMG Labtech).

### Proteomics sample preparation

Cells were lysed in 80 µl lysis buffer containing 5% SDS, 10 mM TCEP and 50 mM TEAB, before boiling for 5 min and sonication using a BioRuptor for 15 cycles of 30 s on and 30 s off. Lysates were then treated with benzonase for 15 min at 37 °C and proteins quantified using the EZQ assay following the manufacturer’s instructions (Thermo Fisher Scientific). Alkylation of proteins was carried out by the addition of iodoacetamide to a final concentration of 20 mM and incubation for 1 h at 20 °C in the dark. Protein lysates were loaded onto S-Trap mini columns (ProtiFi) following the manufacturer’s instructions and proteins digested with trypsin at a protein:trypsin ratio of 20:1. Protein digests were performed at 47 °C for 2 h. Peptides were eluted from mini columns, dried and reconstituted in 1% formic acid.

### Mass spectrometry

Peptides were analyzed using single-shot data-independent acquisition (DIA). For each sample, 200 ng of peptide was injected onto a C18 reverse-phase chromatography system (Vanquish, Thermo Scientific) and electrosprayed into an Astral Orbitrap Mass Spectrometer (Thermo Fisher Scientific) with LC buffers comprising buffer A (0.1% formic acid) and buffer B (80% acetonitrile, 0.1% formic acid). The buffers were used to create a gradient for a run of 30 samples per day where the peptides were eluted from an Aurora Ultimate column (IonOpticks*)* and RAW data were acquired in DIA mode. A scan cycle comprised a full MS scan with an *m/z* range of 380–980, resolution of 240,000, custom automatic gain control target of 500% and a maximum injection time of 3 ms. MS scans were followed by MS/MS DIA scans of isolation windows with widths of 2 Th and an overlap of 0 *m/z*. DIA spectra were recorded with a scan range of 150–2,000 *m/z*, custom automatic gain control target of 500% and a maximum IT of 3 ms. Normalized collision energy was set to 25%. Data for MS scans were acquired in profile mode with MS/MS DIA scan events being acquired in centroid mode.

### Proteomics data analysis

Raw MS data files were searched using Spectronaut (Biognosys) version 19. Raw MS files were searched against a mouse database (Swissprot Trembl, November 2023) with the following parameters: directDIA, false discovery rate set to 1%, protein N-terminal acetylation and methionine oxidation were set as variable modifications, and carbamidomethylation of cysteine residues was selected as a fixed modification. Perseus software^55^ was used to estimate protein copy numbers according to the method described in Wisniewski et al.^56^. Protein copy numbers were normalized to total protein mass per cell.

### In vitro chronic stimulation assay

OT-I splenocytes were activated with anti-CD3 and anti-CD28 antibodies for 24 h and retrovirally transduced as previously described. The following day (day 2), and again 48 h later (day 4), transduced cells were passaged in complete RPMI media supplemented with 100 IU ml^−1^ rhIL-2 and restimulated on plates coated with 5 μg ml^−1^ anti-CD3. Acutely stimulated cells were passaged every 2 days and maintained in complete RPMI media supplemented with 100 IU ml^−1^ rhIL-2. Cells were analyzed by flow cytometry on days 2, 4 and 6. Cytokine polyfunctionality was assessed by intracellular staining following 4-h restimulation on anti-CD3-coated plates in the presence of 5 μg ml^−1^ brefeldin A and 5 μg ml^−1^ monensin.

### JunB CUT&RUN

OT-I T cells were isolated, transduced and subjected to chronic stimulation as previously described. Transduced cells (Thy1.1^+^) were sorted using FACS as previously described and were immediately subjected to CUT&RUN via the CUTANA ChIC/CUT&RUN Kit (EpiCypher) according to the manufacturer’s instructions, with minor modifications. Briefly, 5 × 10^5^ cells per reaction were washed in a buffer containing spermidine and protease inhibitor and then bound to pre-activated concanavalin A beads. Cells were then permeabilized with 0.001% digitonin and incubated overnight at 4 °C with 1 μl of Rabbit anti-JunB (C37F9, Cell Signaling Technologies) in antibody buffer. The following day, micrococcal nuclease fused to proteins A and G (pAG-MNase) was added to the reaction and incubated for 10 min at room temperature. Targeted chromatin digestion and release were activated upon the addition of CaCl_2_, followed by incubation for 2 h at 4 °C. Stop buffer containing *Escherichia coli* spike-in DNA was added to halt the reaction. CUT&RUN-enriched DNA was purified using SPRIselect beads (Beckman Coulter) and quantified using a Qubit Fluorometer (Thermo Fisher Scientific).

DNA libraries were prepared using the CUTANA CUT&RUN Library Prep Kit (EpiCypher) or the NEBNext Ultra II DNA Library Prep Kit for Illumina kit (New England Biolabs) according to the manufacturer’s instructions. Libraries were quality assessed on an Agilent Tapestation using the D1000 ScreenTape (Agilent) and sequenced on a NextSeq 2000 (Illumina) with 100-bp paired-end reads by the Peter MacCallum Cancer Centre Molecular Genomics Core.

### scRNA-seq analysis

Published scRNA-seq data of human TILs were sourced from a public repository (https://zenodo.org/records/5461803)^30^. Downstream analyses were performed using Seurat (v5.1.0) in R v4.3.2. Visualization was performed using ScanPy (v.1.9.1) in Python v3.11.1. Raw gene expression matrices were processed by first removing blacklisted genes as described by the authors^30^. Counts were normalized and scaled, and variable features were found using the SCTransform workflow with regression of mitochondrial and cell cycle-related genes. UMAP plots were generated using the first 25 principal components, and cluster annotation was performed manually according to signature cluster genes.

### RNA-seq and analysis

Single-cell suspensions of tumor-infiltrating Slamf6^+^ and Slamf6^−^ Thy1.1^+^ CD45.1^+^ OT-I cells from B16-OVA-bearing mice, or Thy1.1^+^ OT-I cells following in vitro chronic stimulation as previously described, were purified by FACS. All samples were stored in 40 μl RNA*later* Stabilization Solution (Thermo Fisher) at −80 °C. Samples were processed using the QIAshredder kit (Qiagen) and RNA extracted using the RNeasy Plus Mini Kit (Qiagen) according to the manufacturer’s instructions. RNA libraries were produced using the SMARTer Universal Low Input RNA Kit (Takara) and sequenced on an Illumina NovaSeq 6000 instrument. FASTQ files were quality checked using FastQC and aligned to the GRCm38 (mm10) *Mus musculus* genome assembly using STAR. DESeq2 (v1.42.0)^57^ was used to perform differential gene expression analysis. Further analysis and visualization were completed using R v4.2.2. Principal component analysis was performed using variance stabilizing transformed counts generated using DESeq2. Heat maps of gene expression and gene clustering were performed using the R package pheatmap (v1.0.12). GSEA was performed using the R package fgsea (v1.28.0) with statistical analyses derived from 10,000 permutations.

### Motif enrichment analysis

Genes assigned to the specified clusters through hierarchical clustering of RNA-seq data were used in the analysis. The regions spanning ±2 kb from the TSS of the corresponding genes were analyzed for motif enrichment using HOMER (v5.1)^58^. Motif frequency was calculated by normalizing the absolute number of instances of the indicated motifs in the specified regions by the number of regions analyzed.

### ATAC-seq and analysis

Genome-wide chromatin accessibility measurements were performed using transduced OT-I T cells subjected to chronic stimulation as previously described. Transduced cells (Thy1.1^+^) were sorted using FACS as previously described, and samples were processed for ATAC-seq following Grandi et al. with minor modifications^59^.

In brief, 50,000 sorted cells were washed twice in cold PBS, lysed in ATAC lysis buffer (10 mM Tris-HCl pH 7.5, 10 mM NaCl, 3 mM MgCl_2_, 0.1% Igepal, 0.1% Tween-20, 0.01% digitonin) for 5 min on ice, and nuclei were pelleted by centrifugation at 500*g* for 10 min at 4 °C. Nuclei were resuspended in a 50 µl transposition reaction mix containing 2× TD buffer and 2.5 µl TDE1 transposase (Illumina Tagment DNA Enzyme and Buffer), supplemented with 0.01% digitonin and 0.1% Tween-20, and incubated at 37 °C for 30 min with gentle agitation. DNA was purified using the MinElute PCR Purification Kit (Qiagen). ATAC-seq libraries were generated by PCR amplification with NEBNext High-Fidelity 2× PCR Master Mix (New England Biolabs) and indexed using custom i5/i7 primers. Amplified libraries were purified with the QIAquick PCR Purification Kit (Qiagen), quantified by qPCR (NEBNext Library Quant Kit), and diluted to 10 nM for equimolar pooling. Libraries were sequenced by Novogene on an Illumina NovaSeq X Plus platform with 150-bp paired-end reads.

Processing of FASTQ files was completed as previously described^60^. Briefly, reads were aligned to the GRCm38 (mm10) *Mus musculus* genome assembly using Bowtie2 (ref. ^61^). Mitochondrial, unpaired and unmapped reads were removed using SAMtools. PCR duplicates were removed using Picard and ENCODE blacklist regions removed. Peaks were called using MACS2 with a false discvery rate *q* value < 0.01. Consensus peak sets were generated by combining all peaks across all samples, merging overlapping peaks with the ‘merge’ function in bedtools, and retaining peaks found in more than one sample. Differentially accessible peaks (*q* < 0.1, log_2_(FC) > 1) were identified using DiffBind^62^. Enrichment histograms around BACH2 binding sites were performed using deepTools (v3.5.6)^63^.

### CUT&RUN data processing and analysis

Raw CUT&RUN sequencing data from mouse samples (with K12-MG1655 *E. coli* spike-in) were processed using a series of bash scripts. Adapter and quality trimming were carried out with BBDuk (bbmap v35.19; https://github.com/BioInfoTools/BBMap/). Quality control of raw and trimmed reads was performed using FastQC version 0.11.5. Screening for contamination and alignment rates against the *E. coli* spike-in was performed using FastQ Screen version 0.15.3 (https://www.bioinformatics.babraham.ac.uk/projects/fastqc/). Reads were aligned to the GRCm38 (mm10) *Mus musculus* genome assembly using Bowtie2, followed by SAM/BAM processing using SAMtools version 1.9 and Sambamba (version 0.6.7)^61^. Quantitative normalization of BAM files was performed using the CUT&RUN Greenlist as previously described^64^. Briefly, a curated set of high-confidence CUT&RUN regions (‘greenlist’) was used as an internal reference to correct for technical variability across samples. For each sample i, the total signal within greenlist regions (*S*ᵢ) was computed by summing per-base scores overlapping the greenlist intervals. A scaling factor was then calculated as: scale_ᵢ _= *S*/*S*ᵢ where *S* is the mean greenlist signal across all samples. Per-base scores in each dataset were multiplied by scale_i_ to produce normalized coverage tracks. The adjusted tracks were exported in BigWig and bedGraph formats for downstream analyses and visualization. Enrichment histograms around BACH2 binding sites were performed using deepTools (v3.5.6)^63^.

### Statistical testing

Data were analyzed using statistical tests as indicated in the figure legends. Normality and equal variance were tested before the selection of the statistical test. During tumor experiments, mice were randomized immediately after tumor inoculation and mice were assigned to groups arbitrarily, ensuring consistent starting tumor size across all groups. Researchers and operators were blinded during group allocation, adoptive transfers, tumor measurements, collection and processing of tissues and data analysis. Sample sizes were determined based on prior similar published studies, or prior experience with variability in similar experiments. Samples that experienced technical failures during the execution of procedures or processing were excluded from further analysis.

### Reporting summary

Further information on research design is available in the Nature Portfolio Reporting Summary linked to this article.

## Online content

Any methods, additional references, Nature Portfolio reporting summaries, source data, extended data, supplementary information, acknowledgements, peer review information; details of author contributions and competing interests; and statements of data and code availability are available at 10.1038/s41590-025-02389-z.

## Supplementary information

Reporting Summary

Supplementary Table 1 DEGs in OT-I T cells transduced with BACH2_OE_ or BACH2_DE_ or empty control vectors following chronic stimulation.

Supplementary Table 2 DEGs between intratumoral Slamf6^+^ and Slamf6^−^ OT-I cells transduced with BACH2_OE_, BACH2_DE_ or empty control vectors.

## Source data

Source Data Fig. 1 Statistical source data.

Source Data Fig. 2 Statistical source data.

Source Data Fig. 3 Statistical source data.

Source Data Fig. 4 Statistical source data.

Source Data Fig. 5 Statistical source data.

Source Data Fig. 6 Statistical source data.

Source Data Fig. 7 Statistical source data.

Source Data Fig. 8 Statistical source data.

Source Data Extended Data Fig. 2 Statistical source data.

Source Data Extended Data Fig. 3 Statistical source data.

Source Data Extended Data Fig. 5 Statistical source data.

Source Data Extended Data Fig. 6 Statistical source data.

Source Data Extended Data Fig. 8 Statistical source data.

## Data Availability

RNA-seq and CUT&RUN raw data have been deposited in the European Nucleotide Archive (ENA) database under the accession code ERP182454. Source data are provided with this paper.
