# Supplementary material for: Fine-tuning BACH2 dosage balances stemness and effector function to enhance antitumor T cell therapy
Source: Nat Immunol. 2026 Jan 16;27(3):436–51. doi: 10.1038/s41590-025-02389-z (PMC12956556; doi:10.1038/s41590-025-02389-z)
Supplement: Supplementary file 1 — Reporting Summary [file 41590_2025_2389_MOESM1_ESM.pdf]

nature portfolio

Corresponding author(s): Rahul Roychoudhuri

Last updated by author(s): 21/10/25

Reporting Summary

Nature Portfolio wishes to improve the reproducibility of the work that we publish. This form provides structure for consistency and transparency in reporting. For further information on Nature Portfolio policies, see our Editorial Policies and the Editorial Policy Checklist.

Statistics

For all statistical analyses, confirm that the following items are present in the figure legend, table legend, main text, or Methods section.

n/a	Confirmed	
☐	☒	The exact sample size (*n*) for each experimental group/condition, given as a discrete number and unit of measurement
☐	☒	A statement on whether measurements were taken from distinct samples or whether the same sample was measured repeatedly
☐	☒	The statistical test(s) used AND whether they are one- or two-sided<br>*Only common tests should be described solely by name; describe more complex techniques in the Methods section.*
☒	☐	A description of all covariates tested
☐	☒	A description of any assumptions or corrections, such as tests of normality and adjustment for multiple comparisons
☐	☒	A full description of the statistical parameters including central tendency (e.g. means) or other basic estimates (e.g. regression coefficient) AND variation (e.g. standard deviation) or associated estimates of uncertainty (e.g. confidence intervals)
☐	☒	For null hypothesis testing, the test statistic (e.g. *F*, *t*, *r*) with confidence intervals, effect sizes, degrees of freedom and *P* value noted<br>*Give P values as exact values whenever suitable.*
☒	☐	For Bayesian analysis, information on the choice of priors and Markov chain Monte Carlo settings
☒	☐	For hierarchical and complex designs, identification of the appropriate level for tests and full reporting of outcomes
☒	☐	Estimates of effect sizes (e.g. Cohen's *d*, Pearson's *r*), indicating how they were calculated

Our web collection on statistics for biologists contains articles on many of the points above.

Software and code

Policy information about availability of computer code

Data collection: No software was used.

Data analysis: Published sc-RNAseq data of human TILs was sourced from a public repository (https://zenodo.org/records/5461803). Downstream analyses were performed using Seurat (v5.1.0) in R v4.3.2. Visualization was performed using Scanpy (v.1.9.1) in Python v3.11.1.

FASTQ files were quality-checked using FastQC and aligned to the GRCm38 Mus musculus genome assembly using STAR. DESeq2 (v1.42.0) was used to perform differential gene expression analysis. Further analysis and visualization were completed using R v4.2.2. PCA was performed using variance stabilizing transformed counts generated using DESeq2. Heatmaps of gene expression were created using the R package pheatmap (v1.0.12). GSEA was performed using the R package fgsea (v1.28.0) with statistical analyses derived from 10,000 permutations. Details are described in the Methods section.

Specified gene lists were used in the analysis (see Methods section). The region spanning ± 2kb from the TSS of the corresponding genes were analyzed for motif enrichment using HOMER (v5.1). Motif frequency was calculated by normalizing the absolute number of instances of the indicated motifs in the specified regions by the number of regions analyzed.

Flow cytometry data was analysed using FlowJo v10 (Tree Star Inc.).

For manuscripts utilizing custom algorithms or software that are central to the research but not yet described in published literature, software must be made available to editors and reviewers. We strongly encourage code deposition in a community repository (e.g. GitHub). See the Nature Portfolio guidelines for submitting code & software for further information.

Data

Policy information about availability of data

All manuscripts must include a data availability statement. This statement should provide the following information, where applicable:

- Accession codes, unique identifiers, or web links for publicly available datasets
- A description of any restrictions on data availability
- For clinical datasets or third party data, please ensure that the statement adheres to our policy

RNA-seq and CUT&RUN raw data are deposited in the European Nucleotide Archive (ENA) database under the accession number ERP182454. Source data are provided with this paper.

Research involving human participants, their data, or biological material

Policy information about studies with human participants or human data. See also policy information about sex, gender (identity/presentation), and sexual orientation and race, ethnicity and racism.

Reporting on sex and gender	*Use the terms sex (biological attribute) and gender (shaped by social and cultural circumstances) carefully in order to avoid confusing both terms. Indicate if findings apply to only one sex or gender; describe whether sex and gender were considered in study design; whether sex and/or gender was determined based on self-reporting or assigned and methods used.<br>Provide in the source data disaggregated sex and gender data, where this information has been collected, and if consent has been obtained for sharing of individual-level data; provide overall numbers in this Reporting Summary. Please state if this information has not been collected.<br>Report sex- and gender-based analyses where performed, justify reasons for lack of sex- and gender-based analysis.*
Reporting on race, ethnicity, or other socially relevant groupings	*Please specify the socially constructed or socially relevant categorization variable(s) used in your manuscript and explain why they were used. Please note that such variables should not be used as proxies for other socially constructed/relevant variables (for example, race or ethnicity should not be used as a proxy for socioeconomic status).<br>Provide clear definitions of the relevant terms used, how they were provided (by the participants/respondents, the researchers, or third parties), and the method(s) used to classify people into the different categories (e.g. self-report, census or administrative data, social media data, etc.)<br>Please provide details about how you controlled for confounding variables in your analyses.*
Population characteristics	*Describe the covariate-relevant population characteristics of the human research participants (e.g. age, genotypic information, past and current diagnosis and treatment categories). If you filled out the behavioural & social sciences study design questions and have nothing to add here, write "See above."*
Recruitment	*Describe how participants were recruited. Outline any potential self-selection bias or other biases that may be present and how these are likely to impact results.*
Ethics oversight	*Identify the organization(s) that approved the study protocol.*

Note that full information on the approval of the study protocol must also be provided in the manuscript.

Field-specific reporting

Please select the one below that is the best fit for your research. If you are not sure, read the appropriate sections before making your selection.

[x] Life sciences [ ] Behavioural & social sciences [ ] Ecological, evolutionary & environmental sciences

For a reference copy of the document with all sections, see nature.com/documents/nr-reporting-summary-flat.pdf

Life sciences study design

All studies must disclose on these points even when the disclosure is negative.

Sample size	Sample sizes were determined using variability observed in prior experiments or based on prior experience of sample size requirements. Experiments where technical limitations prevented acquisition of a suitable number of replicates for adequate statistical comparisons, results from multiple identical experiments were pooled together.
Data exclusions	Animals where technical failures prevented procedures being performed adequately (e.g. unsuccessful injections) were excluded from experiments. Technical failure of experiments was objectively determined via inclusion of positive and negative controls where possible. Any sample exclusion was performed according to pre-established criteria to avoid subjective bias.
Replication	The number of independently repeated experiments, and pooling of experimental results, is described in the figure legends. Sample selection for displaying representative examples was performed objectively by identifying the median sample in each group.
Randomization	Sex/aged-matched animals were randomised prior to assignment to control or experimental groups. Acquisition of data from experiments was performed by alternating samples from different groups using identical data acquisition settings to avoid batch effects.
Blinding	Staff performing intravenous injections and tumor measurements were blinded to the experimental groups. Acquisition of data from experiments was performed by alternating samples from different groups using identical data acquisition settings to avoid batch effects. Data analysis was performed in an objective manner by applying identical methodology across all samples.

Reporting for specific materials, systems and methods

We require information from authors about some types of materials, experimental systems and methods used in many studies. Here, indicate whether each material, system or method listed is relevant to your study. If you are not sure if a list item applies to your research, read the appropriate section before selecting a response.

Materials & experimental systems

n/a	Involved in the study	
☐	☒	Antibodies
☐	☒	Eukaryotic cell lines
☒	☐	Palaeontology and archaeology
☐	☒	Animals and other organisms
☒	☐	Clinical data
☒	☐	Dual use research of concern
☒	☐	Plants

Methods

n/a	Involved in the study	
☐	☒	ChIP-seq
☐	☒	Flow cytometry
☒	☐	MRI-based neuroimaging

Antibodies

Antibodies used

Antibody Fluorochrome Clone Supplier Catalogue no. Dilution
anti-CD101 PE-Cy7 Moushi101 eBioscience #25-1011-82 1/500
anti-CD197 (CCR7) BB700 4B12 BD Horizon #566462 1/500
anti-CD279 (PD-1) BV605 29F.1A12 BioLegend #135220 1/1000
anti-CD279 (PD-1) PE-Cy7 RMP1-30 BioLegend #109110 1/1000
anti-CD3 Spark Blue 550 SK7 BioLegend #344852 1/5000
anti-CD4 BUV395 GK1.5 BD Horizon #563790 1/1000
anti-CD44 BV510 IM7 BioLegend #103044 1/1000
anti-CD45.1 BV711 A20 BioLegend #110739 1/500
anti-CD62L BUV737 MEL-14 BD Horizon #612833 1/500
anti-CD69 PE-Cy5 H1.2F3 BioLegend #104510 1/500
anti-CD8 BUV805 53-6.7 BD Horizon #612898 1/1000
anti-CD90.1 (Thy1.1) BUV496 OX-7 BD Horizon #741110 1/1000
anti-CX3CR1 PE/Dazzle 594 SA011F11 BioLegend #149014 1/500
anti-DYKDDDDK PE L5 BioLegend #637310 1/1000
anti-Granzyme B PE QA16A02 BioLegend #372208 1/200
anti-IFNγ BUV737 XMG1.2 BD Horizon #612769 1/200
anti-IL-2 PE/Dazzle 594 JES6-5H4 BioLegend #503840 1/200
anti-Ki67 AF700 16A8 BioLegend #652420 1/2000
anti-KLRG1 BV785 2F1/KLRG1 BioLegend #138429 1/500
anti-Ly108 (Slamf6) PE 330-AJ BioLegend #134606 1/1000
anti-TCF1 AF647 C63D9 Cell Signaling Technology #6709 1/200
anti-TIM-3 BV421 B8.2C12 BioLegend #134019 1/500
anti-TNF BV650 MP6-XT22 BioLegend #506333 1/200
Fixable Viability Dye eFluor 780 eBioscience #65-0865-14 1/1000

Validation

All antibodies have been validated by the manufacturer. Antibody validation information is available for each of the listed antibodies on the relevant manufacturer's website:
anti-CD101 PE-Cy7 Moushi101 eBioscience #25-1011-82: https://www.thermofisher.com/antibody/product/CD101-Antibody-clone-Moushi101-Monoclonal/25-1011-82
anti-CD197 (CCR7) BB700 4B12 BD Horizon #566462: https://www.bdbiosciences.com/en-eu/products/reagents/flow-cytometry-reagents/research-reagents/single-color-antibodies-ruo/bb700-rat-anti-mouse-cd197-ccr7.566462
anti-CD279 (PD-1) BV605 29F.1A12 BioLegend #135220: https://www.biolegend.com/en-gb/products/brilliant-violet-605-anti-mouse-cd279-pd-1-antibody-7648
anti-CD279 (PD-1) PE-Cy7 RMP1-30 BioLegend #109110: https://www.biolegend.com/en-gb/products/pe-cyanine7-anti-mouse-cd279-pd-1-antibody-3612
anti-CD3 Spark Blue 550 SK7 BioLegend #344852: https://www.biolegend.com/en-gb/products/spark-blue-550-anti-human-cd3-antibody-18495
anti-CD4 BUV395 GK1.5 BD Horizon #563790: https://www.bdbiosciences.com/en-gb/products/reagents/flow-cytometry-reagents/research-reagents/single-color-antibodies-ruo/buv395-rat-anti-mouse-cd4.563790
anti-CD44 BV510 IM7 BioLegend #103044: https://www.biolegend.com/en-gb/products/brilliant-violet-510-anti-mouse-human-cd44-antibody-7994
anti-CD45.1 BV711 A20 BioLegend #110739: https://www.biolegend.com/en-gb/products/brilliant-violet-711-anti-mouse-cd45-1-antibody-8925
anti-CD62L BUV737 MEL-14 BD Horizon #612833: https://www.bdbiosciences.com/en-gb/products/reagents/flow-cytometry-reagents/research-reagents/single-color-antibodies/buv737-rat-anti-mouse-cd62l.612833
anti-CD69 PE-Cy5 H1.2F3 BioLegend #104510: https://www.biolegend.com/en-gb/products/pe-cyanine5-anti-mouse-cd69-antibody-266
anti-CD8 BUV805 53-6.7 BD Horizon #612898: https://www.bdbiosciences.com/en-gb/products/reagents/flow-cytometry-reagents/research-reagents/single-color-antibodies-ruo/buv805-rat-anti-mouse-cd8a.612898
anti-CD90.1 (Thy1.1) BUV496 OX-7 BD Horizon #741110: https://www.bdbiosciences.com/en-gb/products/reagents/flow-cytometry-reagents/research-reagents/single-color-antibodies-ruo/buv496-mouse-anti-rat-cd90-mouse-cd90-1.741110
anti-CX3CR1 PE/Dazzle 594 SA011F11 BioLegend #149014: https://www.biolegend.com/en-gb/products/pe-dazzle-594-anti-mouse-cx3cr1-antibody-11908
anti-DYKDDDDK PE L5 BioLegend #637310: https://www.biolegend.com/en-gb/products/pe-anti-dykddddk-tag-antibody-9383
anti-Granzyme B PE QA16A02 BioLegend #372208: https://www.biolegend.com/en-gb/products/pe-anti-human-mouse-granzyme-b-recombinant-antibody-14431

anti-IFNγ BUV737 XMG1.2 BD Horizon #612769: https://www.bdbiosciences.com/en-gb/products/reagents/flow-cytometry-reagents/research-reagents/single-color-antibodies-ruo/buv737-rat-anti-mouse-ifn.612769
anti-IL-2 PE/Dazzle 594 JES6-5H4 BioLegend #503840: https://www.biolegend.com/en-gb/products/pe-dazzle-594-anti-mouse-il-2-antibody-12843
anti-Ki67 AF700 16A8 BioLegend #652420: https://www.biolegend.com/en-gb/products/alexa-fluor-700-anti-mouse-ki-67-antibody-10366
anti-KLRG1 BV785 2F1/KLRG1 BioLegend #138429: https://www.biolegend.com/en-gb/products/brilliant-violet-785-anti-mouse-human-klrg1-mafa-antibody-13682
anti-Ly108 (Slamf6) PE 330-AJ BioLegend #134606: https://www.biolegend.com/en-gb/products/pe-anti-mouse-ly108-antibody-6016
anti-TCF1 AF647 C63D9 Cell Signaling Technology #6709: https://www.cellsignal.com/products/antibody-conjugates/tcf1-tcf7-c63d9-rabbit-mab-alexa-fluor-647-conjugate/6709?srsltid=AfmBOop-aoKcQzY1huxI9q3Fgu6n34wbe6BWEfl1mCq3DgMx3_CnwH3H
anti-TIM-3 BV421 B8.2C12 BioLegend #134019: https://www.biolegend.com/en-gb/products/brilliant-violet-421-anti-mouse-cd366-tim-3-antibody-18197
anti-TNF BV650 MP6-XT22 BioLegend #506333: https://www.biolegend.com/en-gb/products/brilliant-violet-650-anti-mouse-tnf-alpha-antibody-8829
Fixable Viability Dye eFluor 780 eBioscience #65-0865-14: https://www.thermofisher.com/order/catalog/product/65-0865-14

Eukaryotic cell lines

Policy information about cell lines and Sex and Gender in Research

Cell line source(s)
B16-F10 murine melanoma cell line was purchased from American Type Culture Collection. B16-OVA melanoma cell line was kindly provided by Matthew Krummel, who generated the line (Binnewies, M. et al., 2019). MC38-OVA was purhcased from Vitro Biotech. Plat-E cells were purchased from Cell Biolabs.

Authentication
Cell line authentication was performed by the corresponding suppliers. Additional verifications, including cytometry-based analysis, morphological observations, and antigen-mediated cell cytotoxicity assays, yielded expected results. Low-passage stocks were used.

Mycoplasma contamination
Cell lines were screened for mycoplasma contamination and found negative prior to shipment to our facility.

Commonly misidentified lines
(See ICLAC register)
No commonly misidentified cell lines were used.

Animals and other research organisms

Policy information about studies involving animals; ARRIVE guidelines recommended for reporting animal research, and Sex and Gender in Research

Laboratory animals
Details available in the Methods section: OT-I and Ptprca (CD45.1) congenic mice were obtained from the Jackson Laboratory. Bach2tdRFP mice and BACH2-FLAG mice were generated as previously described in Herndler-Brandstetter, D. et al. (2018). Wild-type C57BL/6 mice were purchased from Charles River Laboratories (Wilmington, MA, USA). Experiments were performed with 8- to 12-week-old animals using age- and sex-matched experimental groups. Mice were housed at the University of Cambridge University Biomedical Services (UBS) Gurdon Institute Facility under standard dark/light cycles, temperature and humidity conditions.

Wild animals
No wild animals were used in this study.

Reporting on sex
Female mice was used for adoptive T cell therapy experiments. Both male and female mice were used for all other mice experiments.

Field-collected samples
This study did not involve field-collected samples.

Ethics oversight
Mice were housed at the University of Cambridge University Biomedical Services (UBS) Gurdon Institute Facility. Experiments were conducted in accordance with UK Home Office guidelines and were approved by the University of Cambridge Animal Welfare and Ethics Review Board.

Note that full information on the approval of the study protocol must also be provided in the manuscript.

Plants

Seed stocks
Report on the source of all seed stocks or other plant material used. If applicable, state the seed stock centre and catalogue number. If plant specimens were collected from the field, describe the collection location, date and sampling procedures.

Novel plant genotypes
Describe the methods by which all novel plant genotypes were produced. This includes those generated by transgenic approaches, gene editing, chemical/radiation-based mutagenesis and hybridization. For transgenic lines, describe the transformation method, the number of independent lines analyzed and the generation upon which experiments were performed. For gene-edited lines, describe the editor used, the endogenous sequence targeted for editing, the targeting guide RNA sequence (if applicable) and how the editor was applied.

Authentication
Describe any authentication procedures for each seed stock used or novel genotype generated. Describe any experiments used to assess the effect of a mutation and, where applicable, how potential secondary effects (e.g. second site T-DNA insertions, mosiacism, off-target gene editing) were examined.

ChIP-seq

Data deposition

- [ ] Confirm that both raw and final processed data have been deposited in a public database such as GEO.
- [x] Confirm that you have deposited or provided access to graph files (e.g. BED files) for the called peaks.

Data access links *May remain private before publication.*	RNA-seq and CUT&RUN raw data are deposited in the European Nucleotide Archive (ENA) database under the accession number ERP182454.
Files in database submission	Files correspond to independently generated replicates as described in the Methods section. File names containing 'EV' correspond to empty vector (EV) samples; file names containing 'OE' correspond to BACH2 overexpression (BACH2-OE) samples; file names containing '10Pct' or '5Pct' correspond to BACH2 dosed expression 10% (BACH2DE-10%) and BACH2DE-5% groups respectively.
Genome browser session (e.g. UCSC)	N/A

Methodology

Replicates	Files correspond to independently generated JunB CUT&RUN replicate samples as described in the Methods section. The same pattern of JunB enrichment and peak signal differences between different groups (EV, BACH2OE, BACH2DE) were observed in independent experiments.
Sequencing depth	Details of CUT&RUN procedure, sequencing and analysis are available in the Methods section ('CUT&RUN assay', and 'CUT&RUN data processing and analysis').
Antibodies	Rabbit anti-JunB (Clone: C37F9, Cell Signaling Technologies)
Peak calling parameters	Details of CUT&RUN procedure, sequencing and analysis are available in the Methods section ('CUT&RUN assay', and 'CUT&RUN data processing and analysis').
Data quality	Details of CUT&RUN procedure, sequencing and analysis are available in the Methods section ('CUT&RUN assay', and 'CUT&RUN data processing and analysis').
Software	Details of CUT&RUN procedure, sequencing and analysis are available in the Methods section ('CUT&RUN assay', and 'CUT&RUN data processing and analysis').

Flow Cytometry

Plots

Confirm that:

- [x] The axis labels state the marker and fluorochrome used (e.g. CD4-FITC).
- [x] The axis scales are clearly visible. Include numbers along axes only for bottom left plot of group (a 'group' is an analysis of identical markers).
- [x] All plots are contour plots with outliers or pseudocolor plots.
- [x] A numerical value for number of cells or percentage (with statistics) is provided.

Methodology

Sample preparation	Details available in the Methods section: Single-cell suspensions were blocked with anti-mouse CD16/32 Fc block (BioXCell, 2.4G2) followed by live and dead cell discrimination with Fixable Viability Dye eFluor 780 (Thermo Fisher Scientific). Surface staining was performed for 30 minutes away from light at 4C. Intracellular staining of transcription factors and cytokines was performed overnight following fixation and permeabilization using the eBioscience Foxp3/Transcription Factor Staining Buffer Kit (Invitrogen) and BD Cytofix/Cytoperm Fixation/Permeabilization Kit (BD Biosciences), respectively. Cell counts were obtained using 123count eBeads (Invitrogen).
Instrument	Samples were acquired using a 5-laser Cytek Aurora cytometer.
Software	Flow cytometry data was analysed using FlowJo v10 (Tree Star Inc.).
Cell population abundance	Confirmation of sorted cell populations was obtained via RNA sequencing demonstrating differential gene expression of the marker used for sorting between groups.
Gating strategy	Representative gating strategy is shown in Extended Data Fig. 1.

- [x] Tick this box to confirm that a figure exemplifying the gating strategy is provided in the Supplementary Information.
